# A framework for analyzing EEG data using high-dimensional tests

**DOI:** 10.1093/bioinformatics/btaf109

**Published:** 2025-03-18

**Authors:** Qiuyan Zhang, Wenjing Xiang, Bo Yang, Hu Yang

**Affiliations:** School of Statistics, Capital University of Economics and Business, Beijing 100070, China; School of Information, Central University of Finance and Economics, Beijing 100081, China; School of Preschool Education, Chongqing University of Education, Chongqing 400065, China; School of Information, Central University of Finance and Economics, Beijing 100081, China

## Abstract

**Motivation:**

The objective of EEG data analysis is to extract meaningful insights, enhancing our understanding of brain function. However, the high dimensionality and temporal dependency of EEG data present significant challenges to the effective application of statistical methods. This study systematically addresses these challenges by introducing a high-dimensional statistical framework that includes testing changes in the mean vector and precision matrix, as well as conducting relevant analyses. Specifically, the Ridgelized Hotelling’s $T^{2}$ test (RIHT) is introduced to test changes in the mean vector of EEG data over time while relaxing traditional distributional and moment assumptions. Secondly, a multiple population de-biased estimation and testing method (MPDe) is developed to estimate and simultaneously test differences in the precision matrix before and after stimulation. This approach extends the joint Gaussian graphical model to multiple populations while incorporating the temporal dependency of EEG data. Meanwhile, a novel data-driven fine-tuning method is applied to automatically search for optimal hyperparameters.

**Results:**

Through comprehensive simulation studies and applications, we have obtained substantial evidence to validate that the RIHT has relatively high power, and it can test for changes when the distribution is unknown. Similarly, the MPDe can infer the precision matrix under time-dependent conditions. Additionally, the conducted analysis of channel selection and dominant channel can identify significant channels which play a crucial role in human cognitive ability, such as PO3, PO4, Pz, P4, P8, FT7, and FT8. All findings confirm that the proposed methods outperform existing ones, demonstrating the effectiveness of the framework in EEG data analysis.

**Availability and implementation:**

Source code and data used in the article are available at https://github.com/yahu911/Framework_EEG.

## 1 Introduction

Unlike data gathered through subjective methods, such as focus groups, interviews, and surveys, electroencephalography (EEG) data cannot be consciously influenced (Collura 1993). Tracing these transitions in brain signals, EEG offers a powerful method for studying the effects of external stimuli on neuronal responses. However, brain signals are highly dynamic because they reflect realizations of complex cognitive processes. The dynamic nature results in the collection of EEG data with varying distributions that may evolve the mean vector shift after receiving stimuli. Capturing dynamic changes within EEG data is vital for understanding the brain signal process. For example, determining whether an individual’s neural response occurs in reaction to specific stimuli, such as advertising campaigns, packaging, or designs, provides deeper insights into the effectiveness of these stimuli, and further helps businesses develop more effective marketing strategies that optimize the application of stimuli and improve communication between customers and the stimuli (Khondakar *et al.* 2024). Moreover, capturing the dynamics of individual emotions in daily life has also been widely studied in clinical and well-being contexts, providing a valuable way to regulate emotions and mental health (Damasio and Carvalho 2013), thus facilitating the diagnosis of functional disorders (Kirch *et al.* 2015, Schröder and Ombao 2019, Lu *et al.* 2020).

In statistical analysis, the hypothesis-testing paradigm has garnered significant attention for identifying and verifying sequential changes in EEG data. Statistical hypotheses can be applied not only to test changes in mean vectors but also to evaluate changes in precision matrices, which reflect the conditional dependencies among variables. Various statistical tools, such as the *T*-test, the MANOVA, the regression and the fixed effect model (Ahumada-Mendez *et al.* 2022), have been widely utilized in EEG analysis to uncover meaningful patterns and relationships. However, while current EEG-based brain–computer interfaces (BCIs) often utilize multiple channels to improve brain coverage and enhance source localization, adding more channels can sometimes reduce analysis accuracy and complicate interpretation. This is because while some sensors provide valuable information, others may introduce noise, ultimately degrading the results. To address the challenge in EEG data, although principal component analysis (PCA) (Kambhatla and Leen 1997), independent component analysis (ICA) (Makeig *et al.* 2004, Viola *et al.* 2010) and machine learning (ML) (Saeidi *et al.* 2021) have been proposed to reduce and extract high-dimensional recorded signals, PCA reduces dimensionality by transforming data into uncorrelated principal components; however, it alters the original data structure, thereby reducing interpretability. ICA relies on prior knowledge to select specific EEG channels for analysis, while ML is highly dependent on the sample size. Fortunately, high-dimensional statistical tools provide a solution for analyzing EEG data, providing robust statistical evidence that significantly enhances our understanding of EEG patterns and their underlying mechanisms. Therefore, extending these methods to address the challenges associated with the high dimensionality and temporal dependencies of EEG signals is a worthwhile area of study.

To further elaborate on the problem, let $x_{t}$ denote a *p*-dimensional time series for t∈Z, and let $F_{t}$ represent the distribution function of the random variable $x_{t}$ at time t. The objective is to examine monitoring procedures to identify changes in a parameter $\theta_{t}=\theta (F_{t})$, where θ=θ(F) is a *d*-dimensional parameter of a distribution function F on $R^{d}$ (such as mean, variance, correlation, precision, etc.). Specifically, it aims to establish a decision rule for the hypothesis of a constant parameter, which is defined as:

$$H_{0}:\theta_{1}=\theta_{2}=\ldots =\theta_{n}$$

against the alternative that the parameter changes (once) at some time $\tau_{1},\tau_{2},\ldots ,\tau_{G-1}$ with $1\leq \tau_{1}<\tau_{2}<\ldots <\tau_{G-1}$, i.e.

$$\begin{array}{c} H_{1}:\theta_{1}=\ldots =\theta_{\tau_{1}}=\theta_{1}^{⋆},\theta_{\tau_{1}+1}=\ldots =\theta_{\tau_{2}}=\theta_{2}^{⋆},\ldots =\\ \theta_{\tau_{G-1}+1}=\ldots =\theta_{n}=\theta_{G}^{⋆},\theta_{1}^{⋆}\neq \theta_{2}^{⋆}\neq \ldots \neq \theta_{G}^{⋆} \end{array}$$

The objective of estimating differences among the points $\tau_{1},\ldots ,\tau_{G}$ is to verify and identify significant changes at these points.

Testing for changes in the mean is one of the most commonly used methods in statistical inference and has been widely applied in analyzing EEG data to understand significant shifts in brain activity over time (Kirch *et al.* 2015, Cruz *et al.* 2019, Lu *et al.* 2020). Some of these methods, inspired by CUSUM (Page 1954, Lévy-Leduc and Roueff 2009), focus on establishing a dissimilarity measure based on distributional differences between two intervals. These methods evaluate past and future time series intervals using this measure to verify change points. However, they heavily rely on the assumption that the time series data are generated from a known parametric probability distribution, which may not always be the case, as the underlying probability distribution is sometimes unknown. CUSUM-based methods are highly sensitive to the length of the time series intervals. Longer intervals may delay the detection of change points, while shorter intervals can reduce the method’s effectiveness, potentially leading to false detections or increased noise in the results. Moreover, the dynamic nature and high dimensionality of EEG signals pose significant challenges to the application of traditional statistical testing methods. EEG signals are typically recorded at high sampling rates, ranging from 250 to 1000 samples per second (Hz), across multiple channels, often spanning 64 channels per recording. This high temporal resolution generates a substantial volume of data over time, characterized by significant high dimensionality. The issue of high dimensionality remains a significant challenge, complicating the development of statistical tests for change-point detection. Although numerous high-dimensional testing methods have been proposed in the statistical domain (Jirak 2015, Cho 2016, Wang and Samworth 2018, Wang *et al.* 2022, Jiang *et al.* 2023), these methods have yet to be applied to EEG data analysis. Consequently, their effectiveness and applicability in this context remain unvalidated.

Similarly, understanding changes in dependency patterns among channels before and after the stimulation is also essential because neural among the brain are interconnection (Bullmore and Sporns 2009, Wang *et al.* 2020b, Supriya *et al.* 2023). As a useful tool, graphical models have been successfully applied to infer genetic networks (Yin and Li 2011, Lingjærde *et al.* 2021) and dependency structures among channels in EEG signals (Yaghouby *et al.* 2016, Li and Solea 2018).

Although some advanced methods, such as sparse Gaussian graphical models, have been developed to address the challenges posed by high dimensionality (Friedman *et al.* 2008, Qiao *et al.* 2019). However, their application has predominantly focused on estimating the precision matrix for a single population, with limited attention given to multiple populations. In the context of EEG data analysis, brain signals are highly dynamic as they represent realizations of complex cognitive processes. This dynamism leads to varying distributions across intervals, highlighting the necessity of estimating and testing the dependency structures of different intervals to account for the influence of change points.

Additionally, after verifying the changes, it is also crucial to determine which channels play a significant role in interpreting the stimulus. This can be achieved using high-dimensional statistical tools, such as lasso regression (Tibshirani 1996), which selects important channels without considering channel correlations, and complex network analysis, which identifies dominant channels that serve as influential nodes within the network of channel dependencies. In this study, lasso regression and complex network analysis are integrated into the proposed framework to identify important or dominant channels, providing insights into the brain’s working mechanisms while processing signals.

Considering the dynamic changes and the presence of noisy channels that may occur during EEG analysis, we systematically propose a solution based on high-dimensional statistical methods to address these challenges. Our approach provides a framework for applying high-dimensional testing methods to analyze EEG data effectively. This work makes the following key contributions:

1. Firstly, we propose a framework for EEG data analysis that integrates advanced high-dimensional statistical tools to address challenges arising from high dimensionality. This framework encompasses both testing changes in the mean vector and analyzing variations in the precision matrix, enabling a comprehensive verification of the effects of stimulation.
2. Secondly, we extend the “Ridgelized Hotelling’s *T*^2^ Test (RIHT)” to detect change points pointwise in high-dimensional EEG data to address challenges such as unknown distributions, the selection of time series interval lengths, and the effectiveness of high-dimensional testing for EEG data. This method does not depend on distribution and relaxes the assumptions of the classical four-moment theorem (FMT), requiring only the condition on the first four moments and remaining independent of the underlying random variable distribution.
3. Next, we propose a novel computational algorithm to estimate and infer the precision matrix of EEG data, named as the multiple population de-biased estimation and testing method (MPDe), enabling a deeper understanding of the dependencies among channels before and after stimulation while accounting for the temporal dynamics of EEG signals. This approach is based on a joint Gaussian graphical model with a fused lasso penalty, ensuring structured sparsity and robustness in estimation. Additionally, we introduce a data-driven (DD) method to automatically optimize hyperparameters, enhancing the algorithm’s adaptability and performance.
4. Finally, the face reorganization data have been obtained and used to validate the framework step by step. The application encompasses not only testing changes in mean vectors and precision matrices but also selecting important channels and analyzing dominant channels.

The remainder of this paper is organized as follows. Section 2 provides a comprehensive review of relevant literature on high-dimensional statistical methods for testing both mean vectors and precision matrices. Section 3 presents the proposed RIHT and MPDe methods, including detailed descriptions of their theoretical frameworks, statistical assumptions, asymptotic properties, and corresponding parameter estimation algorithms. Section 4 conducts extensive simulation studies to evaluate the computational efficiency and statistical performance of the proposed methods. Section 5 demonstrates the practical application of the proposed framework through EEG data analysis. Finally, Section 6 concludes the paper with a discussion of the main findings and their implications.

## 2 Related studies

Hypothesis testing can be used to verify sequential changes in EEG data over time in two aspects: first, by examining changes in the mean vector, and second, by analyzing changes in the precision matrix.

### 2.1 Mean vectors testing

Consider a sequence of random vectors $x_{1},x_{2},\ldots ,x_{n}$. Our goal is to verify changes in the sequence at time points $\tau_{1},\tau_{2},\ldots ,\tau_{G-1}$. Specifically, we assume that within each segment $\tau_{g-1}\leq j\leq \tau_{g}$ for g=1,…,G, the random vectors $x_{j}$ follow a common distribution. Here, we define $\tau_{0}=0$ and $\tau_{G}=n$ ensuring that the entire sequence is partitioned into G segments. Additionally, $F_{g}(x)$ represents the population distribution in the gth segment. Thus, the change points $\tau_{0},\tau_{1},\tau_{2},\ldots ,\tau_{G-1},\tau_{G}$ are related to the sample sizes, which are expressed as $n_{1},\ldots ,n_{G}$, and $n_{1}+n_{2}+\ldots +n_{G}=n$. It is worth to note that the sample sizes $n_{1},\ldots ,n_{G}$ are not necessarily equal. Without loss of generality, the samples are written as $x_{j}^{\left(g\right)}$ with the superscript (g) and they are generated from distribution $F_{g}(x)$. The mean vector testing problem focuses the following hypothesis:

(1) $$H_{0}:\mu_{1}=\mu_{2}=\ldots =\mu_{G}$$

 

(2) $$H_{1}:\exists g\;\;\text{such}\;\text{that}\;\;\mu_{g}\neq \mu_{g+1}$$

where $\mu_{g}$ denotes the mean vector of the distribution $F_{g}(x)$ for the gth segment. Testing tools can be utilized to evaluate discrepancies between past and future time series intervals, enabling the precise identification of change in mean. One stream of literature highlights the cumulative sum (CUSUM) method (Page 1954) as a classical approach for segmenting both univariate and multivariate data, applicable to a wide range of changes-in-the-mean models. The CUSUM test evaluates the sample mean before and after a candidate search location to determine whether a specific time point is a change point. Inspired by the CUSUM method, numerous testing approaches have been developed. For instance, Lévy-Leduc and Roueff (2009) enhanced the CUSUM method for high-dimensional network traffic data by incorporating dimension reduction techniques to improve its efficiency and applicability in complex settings. They establish the asymptotic limit distribution with moderate temporal assumptions and spatial assumptions. Considering the sparsity of alternatives, Jirak (2015) introduced a test statistic that constrains the mean vector using the $l_{\infty }$-norm. This approach results in an asymptotic Gumbel distribution. However, this method needs to satisfy two conditions. The one is p→∞ as n→∞ with at most a polynomial rate, and another one is the entries of random vector are independent or weakly dependent. After that, Yu and Chen (2021) relaxed the assumption on the covariance matrix and introduced a uniformly valid test under the condition that p grows sub-exponentially fast in n. Meanwhile, other testing methods, such as the double CUSUM test (Cho 2016), score type test (Husková *et al.* 2007, Kirch *et al.* 2015), an asymptotically minimax estimator (Enikeeva and Harchaoui 2019), change point estimation based on the covariance matrix (Dette *et al.* 2022) are utilized to detect change in high-dimensional data. However, these methods strongly depend on assumptions regarding sparsity, and the divergence rate of dimensionality.

Another stream of literature focuses on robust solution for testing (3) in this context, such as Hotelling’s $T^{2}$ (HT) test proposed by Hotelling (Hotelling 1931), which is affine invariant in nature. Bai and Saranadasa (1996) noted that the exact distribution of classical HT test becomes inapplicable for p→∞, setting due to the singularity of sample covariance matrix $S_{n}$ for high-dimensional data. To address this limitation, Bai and Saranadasa (1996) proposed an asymptotically normally distributed test (ANT) by constructing statistics based on the $l_{2}$-norm of sample mean vector. They demonstrated that the ANT test has greater power than the HT test, particularly when the data dimension is proportionally close to the within-sample degrees of freedom. However, the requirement that p and n must be of the same order is too restrictive, making the method unsuitable for “large p, small n” scenarios. Based on U-statistic, Chen and Qin (2010) proposed a modified version of the ANT method, which allows p to be arbitrarily large and independent of the sample size. Srivastava and Du (2008) proposed a test by modifying the HT statistic, removing all off-diagonal entries of $S_{n}$ and retaining only its diagonal elements. This adjustment makes their test applicable in the p>n setting. The regularized Hotelling’s T^2^ test, a high-dimensional extension of the classical HT test first proposed by Chen *et al.* (2011), enables the high-dimensional mean vector by adding a small perturbation term to $S_{n}$. Their test leverages the ridge idea to construct $l_{2}$-norm based test statistics; however, the proposed method is derived under the assumption of an underlying Gaussian distribution. Further, Li *et al.* (2020) extended the Regularized HT test to the two-sample case, providing its asymptotic distribution under a sub-Gaussian underlying distribution. In this study, we propose the RIHT test, which incorporates a small perturbation term. Our approach focuses on scenarios where the data are distribution-free, eliminating the need for assumptions about the sparsity or the structure of population covariance matrices in high-dimensional setting. Our test can also be adapted for hypothesis testing in scenarios involving multiple change points, assuming a homogeneous covariance structure. Notably, while the proposed test is not strictly affine invariant, it performs favorably compared to the HT statistic for high-dimensional data under minimal conditions.

### 2.2 Precision matrix estimation and testing

We rewrite $x_{j}$ as $x_{j}^{\left(g\right)}$ presenting that $x_{j}$ is generated from gth population. Suppose the p-variate random sample $x_{j}^{\left(g\right)}$ follows a Gaussian distribution. Let G=(V,E) be an undirected graph with a vertex set $V=\{v_{1},v_{2},\ldots ,v_{p}\}$ and an edge set E⊂V×V. Graphs representing complex network structures have ubiquitous value of applications, including models that characterize causal relationships between neurological activity in brain regions, genetic expression across genes, and various other physiological measurements. The precision matrix which represents the conditional independence relationships among the graph’s nodes, reflects the structure of the graph to some extent. In our study, we are not only interested in estimating the precision matrix, which characterizes network structure, but also in understanding how the precision matrix evolves over time.

Most existing studies focus on the point estimation of the precision matrix. The $l_{1}$ penalized normal likelihood estimator, commonly referred to as the $l_{1}$-MLE estimator, is a widely used and effective method for estimating the inverse covariance matrix in Gaussian graphical models. To address the challenges posed by high dimensionality, Yuan and Lin (2007) proposed a method that simultaneously estimates the structure and selects the variables for Gaussian precision graph models. By incorporating the positive definite constraint of the precision matrix, they introduced a maxd*et al*gorithm and demonstrated its effectiveness through theoretical analysis and empirical validation. However, the convergence results of their algorithm are only valid for scenarios where p is fixed and n is large. Later, Banerjee *et al.* (2008) introduced a block coordinate descent algorithm to estimate undirected Gaussian graphical models with sparse structures, capable of handling networks with at least a thousand nodes. Building on this approach, Friedman *et al.* (2008) proposed a simpler algorithm which use the blockwise coordinate descent approach as a launching point and has a remarkably fast computational speeds. Rothman *et al.* (2008) tackled the estimation of precision matrices in high-dimensional settings with a proposed fast iterative algorithm. Additionally, they established a convergence rate of the estimator in the Frobenius norm, allowing both data dimension p and sample size n to grow. Their work highlighted that the convergence rate explicitly depends on the sparsity of the true concentration matrix. Fan *et al.* considered the same normal likelihood model with nonconvex penalties, as detailed in Lam and Fan (2009) and Fan *et al.* (2009). Further contributions to the recovery of precision matrices include work by Meinshausen and Bühlmann (2006), Cai *et al.* (2011), Yuan *et al.* (2019), showcasing a range of advancements in this domain.

Afterwards, scholars began to shift their focus from estimating a single graph to the simultaneous estimation of multiple graphs, particularly for heterogeneous data from different sources. For example, EEG data can be recorded as pre-stimulus and post-stimulus data. Estimating the networks separately for pre-stimulus and post-stimulus data overlooks the common structures shared by the two datasets. Conversely, assuming a single network for both datasets fails to account for the heterogeneity of the data before and after the stimulus. Balancing these considerations is crucial for accurately modeling the network dynamics. Guo *et al.* (2011) studied joint estimation of related precision matrices, where the precision matrices are assumed to be related through a hierarchical structure. Shine *et al.* (2016) proposed the Joint Two-Level Graphical Lasso (JWLGL), applied to fMRI data to explore human brain behavior across functional states. For unbalanced multi-class graphical models, the Joint Adaptive Graphical Lasso (JAGL) procedure is introduced to incorporate a weighted $l_{1}$ penalty (Shan and Kim 2018). Several other methods leveraging regularization approaches to estimate multiple graph structures have been proposed, including the Group Graphical Lasso (Danaher *et al.* 2014), simultaneous clustering and estimation (Hao *et al.* 2018), the Targeted Fused Ridge Estimator (Bilgrau *et al.* 2020), and Gaussian graphical models with latent variables (LVGGM) and PCA-based confounder removal (Wang *et al.* 2023). The Fused Graphical Lasso (FGL) is considered by several statisticians, including Dander *et al.* (2014) and Yang *et al.* (2015). Unlike other methods, FGL borrows information across classes with similar network structures and edge values, making it particularly suitable for EEG data. This feature motivates us to adopt FGL as the penalty in our analysis.

However, few studies focus on hypothesis testing of the precision matrix or provide confidence intervals for the estimators. Some notable works on inference for a single graphical model include Jankova and Van De Geer (2015), both of which use a de-biased method associated with regularization penalties. The ROCKET method is proposed for the edge parameters in transelliptical models under high-dimensional settings without being restricted by Gaussian assumptions (Barber and Kolar 2018). Yu *et al.* (2020) considered pairwise interaction graphical models based on exponential family densities. They introduced a new scoring rule derived from the Hyvärinen scoring method and applied it to hypothesis testing in scenarios with a large number of model parameters. Research on hypothesis testing of the precision matrix for Joint Graph Models (JGM) is even scarcer. Xia *et al.* (2015) proposed a method for testing the equality of two precision matrices, constructing the test statistic based on the maximum of the absolute differences between the matrices. Their method assumes sparsity, which aligns well with genetic data, and leverages sparse information to improve test power. Kim *et al.* (2021) developed a method to compare the equality of two high-dimensional undirected probabilistic graphical models and constructed confidence intervals. Their density-ratio approach avoids direct estimation of the graphs. Wang and Shojaie (2021) addressed the estimation and testing of high-dimensional point process networks, offering a solution for dynamic network inference and hypothesis testing. Although some studies have started to develop hypothesis testing methods for the precision matrix, the research is still relatively limited, l*et al*one its application in EEG data analysis. Considering the temporal dependency inherent in EEG data, we developed an FGL-based precision testing method. This approach not only allows for the estimation of the precision matrix but also provides significance testing for the edges in the graphical model.

## 3 Methodology

### 3.1 Overview

**Figure 1.**
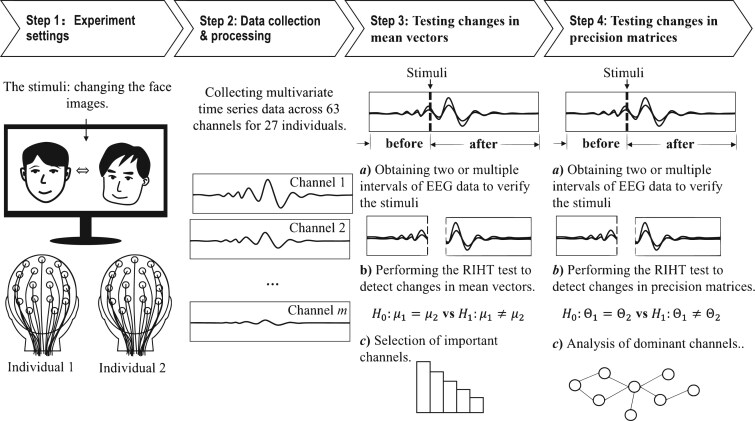
The framework of EEG data analysis using high-dimensional statistical tools which contains four steps: experiment settings, data collection and processing, testing changes in mean vectors and testing changes in precision matrix. (Note: The dotted vertical line marks the stimulus point which is located at time 0 ms.)

The proposed framework for analyzing EEG data in our study is illustrated in Fig. 1. It comprises four steps: experiment settings, data collection and processing, testing changes in mean vectors before and after stimuli, and testing changes in precision matrices before and after stimuli. These last two steps of the framework are performed using high-dimensional statistical tests, which enable the effective examination of changes in the mean vector and precision matrix, even in scenarios where the number of variables significantly exceeds the sample size. We will provide a detailed introduction to them in this section.

Testing changes in mean vectors before and after stimuli represent shifts in the average values of the observed channels, highlighting variations in overall activity or response due to the stimuli. The primary goal of this analysis is to determine whether stimulation has a measurable effect on EEG data and to identify the channels that contribute most significantly to this effect. To achieve this, we introduce the RIHT statistic to test changes in the mean vector, enabling us to evaluate the effect of stimulation and provide statistical evidence to support decision-making. Additionally, we analyze the channels that contribute most significantly to the changes in the mean vector, highlighting those with the greatest impact on the observed variations.

The other part, testing changes in the precision matrix, reflects alterations in the conditional dependencies among channels, revealing how relationships between channels are reorganized in response to the stimuli. Considering both the sparsity and temporal dependency inherent in EEG data, this analysis aims to estimate the precision matrix both before and after stimulation while accounting for these characteristics. The method not only identifies significant elements within the precision matrix but also performs connection analysis to explore the dependencies among channels, ultimately determining which channels dominate these dependencies.

### 3.2 Process of testing changes in mean vectors

#### 3.2.1 The RIHT statistics

It is generally assumed that the cleaned data consists of G independent high-dimensional sample matrices, which are $X_{g}=(x_{1}^{\left(g\right)},\ldots x_{n_{g}}^{\left(g\right)})$, where $x_{1}^{\left(g\right)},x_{2}^{\left(g\right)},\ldots ,x_{n_{g}}^{\left(g\right)},g=1,\ldots ,G$ are random samples independent and identically distributed (i.i.d.) from *g*th *p*-variate Gaussian population with mean $\mu_{g}$, positive definite symmetry covariance $\Sigma_{g}$, dimension p and sample size $n_{g}$. In the following, we denote the total sum of the sample sizes $n_{g}$ as n, i.e. $n=n_{1}+\ldots +n_{G}$. The sample sizes $n_{1},\ldots ,n_{G}$ are not necessarily equal.

Testing the hypothesis on the G-group mean vectors is formulated as

(3) $$H_{0}:\mu_{1}=\mu_{2}=\ldots =\mu_{G}\;vs.\;H_{1}:\text{not}\;H_{0},$$

with the additional assumption that all groups have the equal covariance matrix. (Note: our method can certainly be generalized to the heteroscedasticity case; however, this extension is omitted in the current paper). The classical HT statistic is derived from the Likelihood Ratio Test (LRT) method and is mathematically expressed as follows:

(4) $$\text{HT}=\sum_{g=1}^{G}n_{g}{\left({\bar{X}}_{g}-\hat{\mu }\right)}^{\prime }S_{n}^{-1}({\bar{X}}_{g}-\hat{\mu }),$$

where

(5) $$\begin{array}{l} \hat{\mu }=\frac{1}{n}\sum_{g=1}^{G}\sum_{j=1}^{n_{g}}x_{j}^{\left(g\right)},\;{\bar{X}}_{g}=\frac{1}{n_{g}}\sum_{j=1}^{n_{g}}x_{j}^{\left(g\right)},\\ S_{n}=\frac{1}{e_{1}}\sum_{g=1}^{G}\sum_{j=1}^{n_{g}}\left(x_{j}^{\left(g\right)}-{\bar{X}}_{g}\right){\left(x_{j}^{\left(g\right)}-{\bar{X}}_{g}\right)}^{\prime }, \end{array}$$

and $e_{1}=n-G$, ${\left(\cdot \right)}^{\prime }$ denotes the transpose of a matrix or vector. When the dimension p is larger than $e_{1}$, the HT test becomes undefined, as the sample covariance matrix $S_{n}$ is singular in this high-dimensional setting. To address this challenge, in the case of multiple populations, we propose the RIHT test. This test is designed to overcome the limitations of the HT test in high-dimensional settings. The RIHT statistic is defined as

(6) $$\text{RIHT}=\sum_{g=1}^{G}n_{g}{\left({\bar{X}}_{g}-\hat{\mu }\right)}^{\prime }{\left(S_{n}+\lambda I\right)}^{-1}({\bar{X}}_{g}-\hat{\mu }).$$

Here, we introduce a small perturbation λI to the sample covariance matrix $S_{n}$ to estimate Σ. This approach is analogous to the parameter estimation method used in ridge regression, ensuring that the modified matrix $S_{n}+\lambda I$ is invertible. Note that when $\left\{x_{j}^{\left(g\right)}\right\}$ are Gaussian distributed, the asymptotic null distribution is proposed by Chen *et al.* (2011). Li *et al.* (2020) extend the asymptotic limiting theorem to a sub-Gaussian assumption, and they assert that the test method could be on relaxing the distributional assumptions on the observations. However, the lack of independence between the sample mean and the sample covariance matrix makes it challenging to derive the limiting theory of the RIHT test under arbitrary distributional assumptions for the observations. In our previous work (Zhang *et al.* 2024), we use the exact four-moment theorem (EFMT) to get the central limit theorem of RIHT under a free-of-population distribution of underlying random variables condition requiring a certain number of moments. Suppose samples $\left\{x_{j}^{\left(g\right)}\right\}$ satisfy the following *independent component model*:

(7) $$x_{j}^{\left(g\right)}=T_{g}x_{j}^{\left(g\right)}+\mu_{g},$$

with $\Sigma_{g}=T_{g}T_{g}^{*}$, where $\left\{x_{j}^{\left(g\right)}\right\}$ are i.i.d. random p-variate vectors with independent real standardized components, and denote the components of $x_{j}^{\left(g\right)}$ as $x_{ij}^{\left(g\right)}$ for i=1,…,p. A moment condition on the first four moments is

C1: $E(x_{11}^{\left(g\right)})=0$, $E{\left(x_{11}^{\left(g\right)}\right)}^{2}=1$, $E{\left(x_{11}^{\left(g\right)}\right)}^{3}=0$, and $E{\left(x_{11}^{\left(g\right)}\right)}^{4}=3$.
  From condition C1, we require that the third and fourth moments of $x_{11}^{\left(g\right)}$ match those of a standard real Gaussian distribution. Moreover, some regular conditions are also needed, i.e.
C2: $p,n_{1},\ldots ,n_{g}\to \infty$ such that $\gamma_{n}:=p/n\to \gamma \in (0,\infty )$;
C3: $\tau_{1,p}^{\left(g\right)}\geq \ldots \geq \tau_{p,p}^{\left(g\right)}>0$ are the eigenvalues of $\Sigma_{g}.$ The empirical spectral distribution (ESD) of Σ, defined by $H_{p}(\tau )=\frac{1}{p}\sum_{j=1}^{p}1_{\left[\tau_{j,p}^{\left(g\right)},\infty \right)}(\tau )$, converges as p→∞ to a probability distribution function H(τ) at every continuous point of H, whose support, supp(H), is included in a compact set $\left[h_{1},h_{2}\right]$ with $0<h_{1}\leq h_{2}<\infty$;
C4: $\lim\underset{p\to \infty }{\sup}\tau_{1,p}^{\left(g\right)}<\infty$ and $\lim\underset{p\to \infty }{\inf}\tau_{p,p}^{\left(g\right)}>0$.

The central limit theorem (CLT) of the RIHT test statistic is derived.

Lemma 1. *Suppose that* $\left\{x_{ij}^{\left(g\right)}\right\}$  *and* $\left\{x_{ij}^{\left(g\right)}\right\}$  *are real variables satisfying conditions C1–C4. For* λ>0*, under null hypothesis and* $\Sigma =\Sigma_{1}=\Sigma_{2}=\ldots =\Sigma_{G}$*, we obtain*
 

(8) $$\begin{array}{cc} & T_{\text{RIHT}}(X_{1},\ldots ,X_{G}):=\frac{\sqrt{p}\left(\frac{1}{p}\text{RIHT}-\frac{e_{2}}{p}\text{tr}{\left(S_{n}^{x}+\lambda I\right)}^{-1}\Sigma \right)}{\sqrt{\frac{2e_{2}}{p}\text{tr}{\left(S_{n}^{x}+\lambda I\right)}^{-1}\Sigma {\left(S_{n}^{x}+\lambda I\right)}^{-1}\Sigma }}\\ & \Rightarrow N(0,1), \end{array}$$

*where* $e_{2}=G-1$*, and “*⇒*” denotes the convergence of the distribution.*

When Σ is unknown, we give the estimation theorems of asymptotic mean and asymptotic variance.

Lemma 2. *Under the conditions of Lemma 1, for any* λ>0*, in probability terms*,

(9) $$\sqrt{p}|\frac{1}{p}\text{tr}{\left(S_{n}+\lambda I\right)}^{-1}\Sigma -\Theta_{n}^{\left(1\right)}(\lambda ,\gamma_{n})|\overset{}{\to }0,$$

*and*
 

(10) $$\frac{1}{p}\text{tr}({\left(S_{n}+\lambda I\right)}^{-1}\Sigma {\left(S_{n}+\lambda I\right)}^{-1}\Sigma )-\Theta_{n}^{\left(2\right)}(\lambda ,\gamma_{n})\overset{}{\to }0,$$

*where*
 

(11) $$\Theta_{n}^{\left(1\right)}(\lambda ,\gamma_{n})=\frac{1-\lambda m_{F_{n,p}}(-\lambda )}{1-\gamma_{n}(1-\lambda m_{F_{n,p}}(-\lambda ))},$$

 

(12) $$\begin{array}{c} \Theta_{n}^{\left(2\right)}(\lambda ,\gamma_{n})=\frac{1-\lambda m_{F_{n,p}}(-\lambda )}{{\left(1-\gamma_{n}+\gamma_{n}\lambda m_{F_{n,p}}(-\lambda )\right)}^{3}}\\ -\lambda \frac{m_{F_{n,p}}(-\lambda )-\lambda m_{F_{n,p}}^{\prime }(-\lambda )}{{\left(1-\gamma_{n}+\gamma_{n}\lambda m_{F_{n,p}}(-\lambda )\right)}^{4}}, \end{array}$$

 

(13) $$\begin{array}{c} m_{F_{n,p}}(z)=\frac{1}{p}\text{tr}{\left(S_{n}-zI\right)}^{-1}\;\text{and}\\ m_{F_{n,p}}^{\prime }(z)=\frac{1}{p}\text{tr}{\left(S_{n}-zI\right)}^{-2}. \end{array}$$

Since then, we have completed the mean test without any restriction on sample size, dimension, distribution information of population, and the information of Σ. If we are confident in the estimation of the precision matrix Θ or Σ is known, we directly use $T_{\text{RIHT}}(X_{1},\ldots ,X_{G})$ to execute the hypothesis test without estimating the asymptotic mean and asymptotic variance. Finally, as we can see from Lemma 1 and Lemma 2, we do not require that the distribution of the sample is Gaussian distributed. The conclusion is general. Of course, we can suppose that the neural activity data of brain regions is generated by a multivariate Gaussian distribution, in which case the proposed RIHT test is still feasible.

#### 3.2.2 Change validation procedure

Following with the idea of CUSUM-based methods (Page 1954, Lévy-Leduc and Roueff 2009), which involves defining a dissimilarity measure based on the distribution difference between two intervals, we test changes in mean vectors before and after stimuli by the RIHT statistic. This approach can also be extended to verify changes across multiple intervals. In this case, we first compare the EEG data across multiple intervals at change points to determine whether there are significant differences. If a significant difference is detected, such as when the RIHT statistic exceeds the threshold, we then test the EEG data between any two adjacent intervals to identify the specific time points where differences exist. Therefore, the testing procedure consists of two steps: (i) a multi-interval test to detect overall changes and (ii) a point-by-point test between two intervals to pinpoint specific change points. The procedure is described in Algorithm 1.

Algorithm 1
Algorithm of testing changes in mean vector.

**Require:** Loading dataset $X=(X_{1},\ldots ,X_{G})$.

**Ensure:**

1: Initialization: dividing **X** into G parts, such as $x_{1}^{\left(g\right)},x_{2}^{\left(g\right)},\ldots ,x_{n_{g}}^{\left(g\right)}$ for g=1,…,G;

2:  Performing a multi-interval test by the RIHT testing to detect overall changes, such as:

$$H_{0}:\mu_{1}=\mu_{2}=\ldots =\mu_{G}\;\text{VS}\;H_{1}:\exists g\;\text{such}\;\text{that}\;\mu_{g}\neq \mu_{g+1};$$

3: If $|T_{\text{RIHT}}(X_{1},\ldots ,X_{G})|>1.96$ then performing a point-by-point test among intervals to pinpoint specific change points.

4: For g=1,…,G:

5: Performing a point-by-point test between two intervals to pinpoint specific change points, such as:

   $H_{0}:\mu_{g}=\mu_{g+1}\;\text{VS}\;H_{0}:\mu_{g}\neq \mu_{g+1};$

6: If $|T_{\text{RIHT}}(X_{g},X_{g+1})|>1.96$ then the time point g is recorded as a significant change point.

7: Repeat until finishing testing.

#### 3.2.3 Channels analysis

Current EEG-based BCIs often use multiple channels to enhance brain coverage and source localization. However, adding more channels can decrease analysis accuracy and complicate interpretation, as some sensors provide valuable information while others introduce noise, degrading results. Extracting relevant features from EEG signals is a key challenge in BCIs systems (Meziani *et al.* 2019), particularly due to the high-dimensional nature of the data and its intrinsic structure, including channel location and time epoch (Puk *et al.* 2022).

Assuming the stimulation occurs at time j for a given sample, we define an indicator variable Y, whose length matches that of the EEG sample. The elements of Y are set to 0 for time points before the stimulation and to 1 for time points after the stimulation. To identify dominant channels in our study, we use lasso logistic regression, which facilitates variable selection. Consider the samples S=(Y,X) with n observations where Y is the response n × 1 vector ${\left(Y_{1},Y_{2},\ldots ,Y_{n}\right)}^{T}$ and X is a n × p design matrix with p predictors $X=(X_{1},X_{2},\ldots ,X_{p})$. The predictors are channels. All of the predictor $\left(X_{1},X_{2},\ldots ,X_{p}\right)$ and Y are centered and standardized before performing the Lasso regression procedure respectively. The Lasso is the regularization technique for simultaneous estimation and variable selection (Tibshirani 1996), defined as

(14) $$\hat{\beta }=\arg\underset{\beta }{\min}\frac{1}{n}{\left\|Y-X\beta \right\|}^{2}+\lambda {\left\|\beta \right\|}_{1}$$

where the 1×p coefficients vector $\beta ={\left(\beta_{1},\beta_{2},\ldots ,\beta_{p}\right)}^{T}$ and its estimates are $\hat{\beta }$ respectively, and λ is nonnegative regularization parameter. The second term $\|\beta {\|}_{1}$ in 14 is the so-called “lasso penalty” ($l_{1}$ penalty), which is the sum of absolute value of coefficients of predictors. If we change the sum of absolute value of coefficients of predictors to the sum of square of coefficient of predictors, the penalty is so-called the “ridge penalty” ($l_{2}$ penalty), noted as $\|\beta {\|}^{2}$ or $\sum \beta^{2}$.

### 3.3 Process of testing changes in precision matrices

#### 3.3.1 The fused graphical lasso

Gaussian Graph Model (GGM) is an application of the probability graph model, which is a valid tool to describe the conditional independence relationships among a set of random variables. Thus, it can be applied to conduct functional connectivity analysis in the context of EEG analysis. We suppose that $x_{j}^{\left(g\right)}$ is Gaussian distributed. Let G=(V,E) be an undirected graph with a vertex set $V=\{v_{1},v_{2},\ldots ,v_{p}\}$ and an edge set E⊂V×V. $x_{1,j}^{\left(g\right)},x_{2,j}^{\left(g\right)},\ldots ,x_{p,j}^{\left(g\right)}\in V$ are the vertices of a graph G, and the connectivity or structure of graph G is determined by the conditional independence of $x_{i_{1},j}^{\left(g\right)}$ and $x_{i_{2},j}^{\left(g\right)}$ given all other variables, where $x_{i,j}^{\left(g\right)}$ denotes the ith component of $x_{j}^{\left(g\right)}$. If $x_{i_{1},j}^{\left(g\right)}$ and $x_{i_{2},j}^{\left(g\right)}$ are conditionally independent, then the (i,j)-entry of precision matrix Θ is zero; otherwise, (i,j)-entry is not equal to zero. After that, the connectivity of the graph can be inferred based on nonzero entries of $\Theta_{g}:=\Sigma_{g}^{-1}$, also known as the inverse covariance matrix, see details Tsai *et al.* (2022). Meanwhile, it is of great significance to estimate elements of the precision matrix and perform a hypothesis on it. To estimate the precision matrices for multiple populations, we use the JGM, which minimizes the log-likelihood

(15) $$\begin{array}{cc} & \left\{{\hat{\Theta }}_{g}\right\}\\ = & \arg\min_{\left\{\Theta_{g}\in S^{++}\right\}}\sum_{g}\{\text{tr}(S_{n_{g}}\Theta_{g})-\text{log}\;\det(\Theta_{g})\}\\ & +P(\{\Theta_{g}\}). \end{array}$$

where $S_{n_{g}}=\frac{1}{n_{g}}X_{g}X_{g}^{\prime }$, $P(\{\Theta_{g}\})$ denotes the penalty function, $\left\{{\hat{\Theta }}_{g}\right\}$ are the minimizers of (15), and we optimize (15) over symmetric positive definite matrices set $S^{++}$. Note that the estimation of the JGM largely relies on the penalty, where the penalty reflects the information on the assumed structure of population precision matrices and their connection. Taking into account the temporal dependency inherent in EEG data, the connectivity between multiple channels exhibits a seamless evolution over time. Therefore, we introduce the FGL penalty to constrain the network structure and facilitate a smooth evolution in estimation. The JGM with FGL is defined as

(16) $$P(\{\Theta_{g}\})=\lambda_{1}\sum_{g=1}^{G}||{\left(\Theta_{g}\right)}^{-}|{|}_{1}+\lambda_{2}\sum_{g<g^{\prime }}||{\left(\Theta_{g}-\Theta_{g^{\prime }}\right)}^{-}|{|}_{1},$$

where $\lambda_{1}$ and $\lambda_{2}$ are nonnegative regularization parameter, ${\left(\Theta_{g}\right)}^{-}$ represents the matrix obtained by setting the diagonal elements of $\left(\Theta_{g}\right)$ to zero, and $||\cdot |{|}_{1}$ denotes $l_{1}$-norm of a vector or matrix. The first term in (16) is the classical lasso penalty, which shrinks the coefficients toward 0 as $\lambda_{1}$ increases, and the fused lasso penalty on ${\left(\Theta_{g}-\Theta_{g^{\prime }}\right)}^{-}$ encourages the elements of ${\hat{\Theta }}_{1},\ldots ,{\hat{\Theta }}_{G}$ to have the similar network structure across different populations.

Note: (i) Performing hypothesis tests and constructing confidence intervals are challenging due to the use of implicit penalty estimators. Until now, the majority of statistical approaches have focused on the statistical inference of graphical models within a single graph framework. In contrast, only a few studies have delved into statistical inference for multiple graphs or the JGM. (ii) With the imposition of penalties, the estimation becomes biased. It will have an impact on the inference. Therefore, we need to propose a de-bias estimator for FGL, which incorporates point estimation of precision matrices while simultaneously conducting hypothesis testing.

#### 3.3.2 Estimation procedure

To achieve an unbiased estimator for FGL, we have improved a de-biasing method aimed at mitigating the bias introduced by the penalty. The method is proposed by Janková and van de Geer (2018), and they investigated a de-biasing technology to obtain a consistent estimator with a known distribution. However, they only get an estimation of the precision matrix of a graph. We extend the de-bias method to estimate the JGM with FGL, i.e.

$${\tilde{\Theta }}_{g}=2{\hat{\Theta }}_{g}-{\hat{\Theta }}_{g}S_{n_{g}}{\hat{\Theta }}_{g},$$

where ${\hat{\Theta }}_{g}$ is derived from (15) by algorithm for JGM with FGL.

#### 3.3.3 Testing procedure

To conduct hypothesis testing on the precision matrix, our focus lies on the following multiple linear hypotheses:

(17) $$H_{0}:a_{1}\Theta_{1;ij}^{0}+\ldots +a_{G}\Theta_{G;ij}^{0}=0\;vs.\;H_{1}:\text{not}\;\;H_{0},$$

where $a_{1},\ldots ,a_{G}$ are known constants, $\Theta_{g;ij}^{0}$ is the (i,j)-entry of $\Theta_{g}^{0}$. Then, we propose the test statistics as

(18) $$T_{ij}=a_{1}{\tilde{\Theta }}_{g;ij}+\ldots +a_{G}{\tilde{\Theta }}_{G;ij},$$

whose asymptotic null distribution is

(19) $$\sqrt{n}[T_{ij}-\Theta_{ij}^{0}]\to_{D}\;N(0,\sigma_{ij}^{2}),$$

where $f(x_{1},\ldots ,x_{G})=a_{1}x_{1}+\ldots +a_{G}x_{G}$, $T_{ij}=f\left({\tilde{\Theta }}_{1;ij},\ldots ,{\tilde{\Theta }}_{G;ij}\right)$, and $\Theta_{ij}^{0}=f\left(\Theta_{1;ij}^{0},\ldots ,\Theta_{G;ij}^{0}\right)=0$. The asymptotic variance $\sigma_{ij}$ in (19) is unknown, so to construct confidence intervals we use a consistent estimator

(20) $${\hat{\sigma }}_{ij}^{2}=f_{v}([{\tilde{\Theta }}_{1;ii}{\tilde{\Theta }}_{1;jj}+{\tilde{\Theta }}_{1;ij}^{2}],\ldots ,[{\tilde{\Theta }}_{G;ii}{\tilde{\Theta }}_{G;jj}+{\tilde{\Theta }}_{G;ij}^{2}]),$$

where $f_{v}(x_{1},\ldots ,x_{G})=a_{1}^{2}x_{1}+\ldots +a_{G}^{2}x_{G}$. Then, we get the limiting result of the proposed test under null hypothesis

(21) $$T_{\mathrm{MPDe};ij}(X_{1},\ldots ,X_{G}):=\frac{\sqrt{n}[T_{ij}-\Theta_{ij}^{0}]}{{\hat{\sigma }}_{ij}}\to_{D}\;N(0,1).$$

#### 3.3.4 Algorithm of estimating parameters in the fused graphical lasso

To solve the optimization problem (15) with FGL penalty, we first rewrite it as

(22) $$\begin{array}{cc} & \text{minimize}\;\sum_{g}\{\text{tr}(S_{n_{g}}\Theta_{g})-\text{log}\;\det(\Theta_{g})\}+P(\{\Theta_{g}\})\\ & \text{subject}\;\text{to}\;\Theta_{g}=Z^{\left[g\right]},\;\Theta_{g}-\Theta_{g+1}=\Delta^{\left[g\right]}. \end{array}$$

where $Z^{\left[g\right]}$ and $\Delta^{\left[g\right]}$ are the slack variables. The augmented Lagrangian for this problem is

(23) $$\begin{array}{l} \;\;\;\;L\left(\left\{\Theta_{g}\},\{Z^{\left[g\right]}\},\{\Delta^{\left[g\right]}\},\{V^{\left[g\right]}\},\{U^{\left[g\right]}\right\}\right)\\ =\sum_{g}\{\text{tr}(S_{n_{g}}\Theta_{g})-\text{log}\;\det(\Theta_{g})\}\\ +\sum_{g=1}^{G}\{\lambda_{1}||{\left(Z^{\left[g\right]}\right)}^{-}|{|}_{1}\\ +(\rho_{1}/2)||{\left(\Theta_{g}-Z^{\left[g\right]}+V^{\left[g\right]}\right)}^{-}|{|}_{F}^{2}\\ -(\rho_{1}/2)||{\left(V^{\left[g\right]}\right)}^{-}|{|}_{F}^{2}\}\\ +\sum_{g=1}^{G-1}\{\lambda_{2}||{\left(\Delta^{\left[g\right]}\right)}^{-}|{|}_{1}\\ +(\rho_{2}/2)||{\left(\Theta_{g}-\Theta_{g+1}-\Delta^{\left[g\right]}+U^{\left[g\right]}\right)}^{-}|{|}_{F}^{2}\\ -(\rho_{2}/2)||{\left(U^{\left[g\right]}\right)}^{-}|{|}_{F}^{2}\}, \end{array}$$

where $V^{\left[g\right]}$ and $U^{\left[g\right]}$ are the scaled dual variable associated with the constraint, and $\rho_{1}$ and $\rho_{2}$ are the positive penalty parameters, $||\cdot |{|}_{F}$ denotes Frobenius norm. If g=1, denotes

(24) $$\begin{array}{cc} & Y^{\left[1\right]}(V^{\left[1\right]},Z^{\left[1\right]},U^{\left[1\right]},\Delta^{\left[1\right]})\\ = & S_{n_{1}}+\rho_{1}\left(V^{\left[1\right]}-Z^{\left[1\right]}\right)+\rho_{2}\left(U^{\left[1\right]}-\Delta^{\left[1\right]}-\Theta_{2}\right); \end{array}$$

if 1<g<G, denotes

(25) $$\begin{array}{cc} & Y^{\left[g\right]}(V^{\left[g\right]},Z^{\left[g\right]},U^{\left[g\right]},\Delta^{\left[g\right]})\\ = & S_{n_{g}}+\rho_{1}\left(V^{\left[g\right]}-Z^{\left[g\right]}\right)\\ & -\rho_{2}\left(U^{\left[g-1\right]}-\Delta^{\left[g-1\right]}+\Theta_{g-1}\right)\\ & +\rho_{2}\left(U^{\left[g\right]}-\Delta^{\left[g\right]}-\Theta_{g+1}\right); \end{array}$$

otherwise,

(26) $$\begin{array}{cc} & Y^{\left[G\right]}(V^{\left[G\right]},Z^{\left[G\right]},U^{\left[G\right]},\Delta^{\left[G\right]})\\ = & S_{n_{G}}+\rho_{1}\left(V^{\left[G\right]}-Z^{\left[G\right]}\right)\\ & -\rho_{2}\left(U^{\left[G-1\right]}-\Delta^{\left[G-1\right]}+\Theta_{G-1}\right). \end{array}$$

A piecewise function $m_{\alpha }(z)$ of a variable z with parameter α is defined as

(27) $$m_{\alpha }(z)=\{\begin{array}{c} z+\alpha ,\;z<-\alpha \\ 0,\;|z|<\alpha \\ z-\alpha ,\;z>\alpha , \end{array}$$

and the function $M_{\alpha }(Z)$ of a matrix Z returns a matrix, i.e. $M_{\alpha }(Z)={\left(m_{\alpha }(Z_{ij})\right)}_{i,j}.$ Let $Y^{\left[g\right]}$ be the off-diagonal matrix of $Y^{\left[g\right]}$. The updated rules are

(28) $$\Theta_{g;(i_{t})}\leftarrow \{\begin{array}{c} \frac{-Y_{\left(i_{t}-1\right)}^{\left[g\right]}+\sqrt{\Xi +4(\rho_{1}+\rho_{2})I}}{2(\rho_{1}+\rho_{2})}\;g=1,G\\ \frac{-Y_{\left(i_{t}-1\right)}^{\left[g\right]}+\sqrt{\Xi +4(\rho_{1}+2\rho_{2})I}}{2(\rho_{1}+2\rho_{2})}\;1<g<G. \end{array}$$

 

(29) $$Z_{\left(i_{t}\right)}^{\left[g\right]}\leftarrow {\left(M_{\frac{\lambda_{1}}{\rho_{1}}}(\Theta_{g;(i_{t})}+V_{\left(i_{t}-1\right)}^{\left[g\right]})\right)}^{-}$$

 

(30) $$\Delta_{\left(i_{t}\right)}^{\left[g\right]}\leftarrow {\left(M_{\frac{\lambda_{2}}{\rho_{2}}}(\Theta_{g;(i_{t})}-\Theta_{g+1;(i_{t})}+U_{\left(i_{t}-1\right)}^{\left[g\right]})\right)}^{-}$$

 

(31) $$V_{\left(i_{t}\right)}^{\left[g\right]}\leftarrow {\left(V_{\left(i_{t}-1\right)}^{\left[g\right]}+\Theta_{g;(i_{t})}-Z_{\left(i_{t}\right)}^{\left[g\right]}\right)}^{-}$$

 

(32) $$U_{\left(i_{t}\right)}^{\left[g\right]}\leftarrow {\left(U_{\left(i_{t}-1\right)}^{\left[g\right]}+\Theta_{g;(i_{t})}-\Theta_{g+1;(i_{t})}-\Delta_{\left(i_{t}\right)}^{\left[g\right]}\right)}^{-},$$

where $i_{t}$ is the iteration of the algorithm, and $\Xi ={\left(Y_{\left(i_{t}-1\right)}^{\left[g\right]}\right)}^{\prime }Y_{\left(i_{t}-1\right)}^{\left[g\right]}$. From the analysis above, we note that the positive definiteness constraint on the precision matrices is naturally enforced by the update (28). In our algorithm, the stopping criterion is

(33) $$\frac{\sum_{g=1}^{G}||{\hat{\Theta }}_{g;(i_{t})}-{\hat{\Theta }}_{g;(i_{t}-1)}|{|}_{1}}{\sum_{g=1}^{G}||{\hat{\Theta }}_{g;(i_{t}-1)}|{|}_{1}}\leq \varepsilon ,$$

where $\varepsilon =10^{-9}$, and ${\hat{\Theta }}_{g;(i_{t})}$ denotes the estimate of $\Theta_{g;(i_{t})}$ in the $i_{t}$th step of the algorithm. Algorithm 2 represents the iterative process for multiple populations FGL model.

Algorithm 2
Estimating parameters of the fused graphical lasso.

**Require:** Loading dataset $X_{g}$, and the nonnegative parameters $\lambda_{1}$, $\rho_{1}$, $\lambda_{2}$, and $\rho_{2}$.

**Ensure**

1:  Initialization: ${\hat{\Theta }}_{g;(0)}={\left(S_{n_{g}}+0.1I\right)}^{-1}$, $Z_{\left(0\right)}^{\left[g\right]}={\hat{\Theta }}_{g;(0)}$, $\Delta_{\left(0\right)}^{\left[g\right]}={\hat{\Theta }}_{g;(0)}-{\hat{\Theta }}_{g+1;(0)}$, $V_{\left(0\right)}^{\left[g\right]}=1_{p}1_{p}^{\prime }$, and $U_{\left(0\right)}^{\left[g\right]}=1_{p}1_{p}^{\prime }$;

2:  While iteration ¡ maximize iteration:

3:  In the $i_{t}$th iteration, given the estimated ${\hat{\Theta }}_{g;(i_{t})}$, updating (28)-(32);

4:  Repeat until convergence.

#### 3.3.5 Algorithm of tuning hyperparameters in the fused graphical lasso

Now, we discuss how to select the values of hyperparameters $\lambda_{1}$, $\lambda_{2}$, $\rho_{1}$ and $\rho_{2}$ for the proposed method in (23). Some indices, such as AIC (Akaike Information Criterion), are highly beneficial for selecting tuning parameters to estimate prediction error and compare different models. Some penalized methods (Yang and Yi 2015) obtained tuning parameters by fixing a grid of values for tuning parameters, such as (0,0.01,0.01,1,10,100), and then chose optimal tuning parameters from the model which holds the smallest error. However, determining the appropriate range of tuning parameters and selecting the optimal value for tuning parameters becomes challenging when dealing with numerous hyper-parameters. In the proposed method, the inclusion of four hyper-parameters in the model makes it challenging to determine the suitable range of the grid for all four parameters. In practice, the setting of the search space depends on prior knowledge, but often we lack such knowledge. Another challenge is that the computational complexity of searching hyper-parameters increases exponentially as the number of hyper-parameters grows. For example, given that the search space for each hyper-parameters comprises 10 parameters, the total number of hyper-parameters is $10^{4}$ for the proposed method. These challenges will hinder our ability to find the best combination of hyper-parameters for the proposed method, thereby impeding its application.

To settle this issue, inspired by previous study (Shrivastava *et al.* 2020), we have introduced a DD method for selecting optimal hyper-parameters through deep learning without prior information. The previous study frames the process of estimating the precision matrix as a sparse graph recovery problem, using l1-regularized maximum likelihood estimation. It selects optimal parameters solely based on the data. In contrast, considering the temporal dependency, the fused lasso penalty is involved in the optimization problem in (22). The augmented Lagrangian method is introduced to estimate the unknown precision matrix both before and after the change point. The hyper-parameters $\lambda_{1}$, $\lambda_{2}$, $\rho_{1}$, and $\rho_{2}$ are replaced by problem dependent neural networks: $\Lambda_{nn}$ and $\rho_{nn}$. These neural networks have a minimal number of parameters due to the low input dimensions(from 4 to 7 for $\Lambda_{nn}$ or $\rho_{nn}$) and output a single value.

Specifically, the first neural network $\Lambda_{nn}$ is utilized to update $\lambda_{1}$ in Equation (23). The inputs to this neural network include $Z_{g}$, $\Theta_{g}$ and the initial or last iteration value of $\lambda_{1}$. The second neural network $\Lambda_{nn}$ updates $\lambda_{2}$ using $\Delta_{g}$, $\Theta_{g}$, $\Theta_{g+1}$ and the initial or last iteration value of $\lambda_{2}$ as inputs. Similarly, two neural networks $\rho_{nn}$ are used to update $\rho_{1}$ and $\rho_{2}$ respectively. The inputs of first $\rho_{nn}$ are $\Sigma_{g}$, $\Theta_{g}$, $Z_{g}$ and $\rho_{1}$, while the inputs of second are $\Theta_{g}$, $\Theta_{g+1}$, $\Delta_{g}$ and $\rho_{2}$. Algorithm 3 is described as follows. The architectural details are illustrated in the previous study (Shrivastava *et al.* 2020). Both $\Lambda_{nn}$ and $\rho_{nn}$ represent single-layer fully connected neural networks, which can be essentially regarded as linear transformations.

Algorithm 3
Tuning hyper-parameters through the data-driven (DD) method

**Require**: Loading dataset $X_{g}$ for g=1,2,…,G

**Ensure**: Choosing the optimal hyper-parameters $\lambda_{1}$, $\lambda_{2}$, $\rho_{1}$ and $\rho_{2}$

1:  Initialization: $\lambda_{1;(0)}$, $\lambda_{2;(0)}$, $\rho_{1;(0)}$ and $\rho_{2;(0)}$;

2:  While iteration ¡ maximize iteration:

3:  In the $i_{t}$th iteration, the following processes are executed.

4:  Updating $\lambda_{1;(i)}\leftarrow \Lambda_{nn}(\sum_{g=1}^{G}\|Z_{g;(i)}-\Theta_{g;(i)}{\|}_{F}^{2},\lambda_{1;(i)})$

5:  Updating $\lambda_{2;(i)}\leftarrow \Lambda_{nn}(\sum_{g=1}^{G-1}\|\Delta_{g;(i)}-(\Theta_{g;(i)}-\Theta_{g+1;(i)}){\|}_{F}^{2},\lambda_{2;(i)})$;

6:  Fixed hyper-parameters, updating $\Theta_{g;(i)}$ for g=1,2,…,G;

7:  Updating $\rho_{1;(i)}\leftarrow \rho_{nn}(\Sigma_{g;(i)},\Theta_{g;(i)},Z_{g;(i)},\rho_{1;(i)})$;

8:  Updating $\rho_{2;(i)}\leftarrow \rho_{nn}(\Theta_{g;(i)}-\Theta_{g+1;(i)},\Delta_{g;(i)},\rho_{2;(i)})$;

9:  Updating $Z_{g;(i)}$, $\Delta_{g;(i)}$, $V_{g;(i)}$ and $U_{g;(i)}$ for g=1,2,…,G;

10:  Repeat until convergence.

## 4 Simulation studies

To evaluate the effectiveness of the proposed high-dimensional testing statistics, particularly in their ability to accurately detect differences within EEG data, we utilize simulation data in this section to validate and demonstrate their performance.

### 4.1 Simulation for testing changes in mean vectors

Firstly, we examine the power of the proposed test comparing other methods $T_{\mathrm{FHW}}$ test (Fujikoshi *et al.* 2004), $T_{S}$ test (Schott 2007), $T_{SK}$ test (Srivastava and Kubokawa 2013), and $T_{\mathrm{HBWW}}$ test (Hu *et al.* 2017). We set p=63, corresponding to the number of channels in the EEG data collected during the experiments. The sample size are set to $\left(n_{1},n_{2})=(100,20\right)$ and $\left(n_{1},n_{2})=(20,20\right)$, which closely align with the characteristics of the experimental data. We choose the observation $x_{j}^{\left(g\right)}$ from the *independent component model*
 

(34) $$x_{j}^{\left(g\right)}=T_{g}x_{j}^{\left(g\right)}+\mu_{g},\;g=1,2.$$

We set $x_{ij}^{\left(g\right)}$ satisfying following model,

**Model I. (Gaussian)**  $x_{ij}^{\left(g\right)}$ takes the standard normal distribution;
**Model II. (Mixed linear)** the random variable $x_{ij}^{\left(g\right)}$ is generated with
  (35) $$x_{ij}^{\left(g\right)}=\tilde{m}z_{1}+\hat{m}z_{2},$$
  where $\tilde{m}=0.7827,\hat{m}=0.6224$. The random variable $z_{1}$ is generated from a uniform distribution on the interval $\left(-\sqrt{3},\sqrt{3}\right)$. The variable $z_{2}$ is independent of $z_{1}$ and follows a distribution with the density
  (36) $$f(z)=\{\begin{array}{cc} \frac{\sqrt{2}}{2}e^{-\sqrt{2}z} & \text{if}\;z>0,\\ \frac{\sqrt{2}}{2}e^{\sqrt{2}z} & \text{if}\;z<0. \end{array}$$

To compare the empirical power of the proposed test with other tests, we evaluate the following alternative case.

$\mu_{1}=0_{p}$ , where $0_{p}$ is a p-dimensional column vector with all elements being 0. Denote $\mu_{2j}={\left(-1\right)}^{j}\kappa s^{*}\nu_{j}$ as the jth component of mean vector $\mu_{2}$, where $\nu_{j}∼N(0,1)$. Here, κ is a constant ranging from 1 to 20, $s^{*}$ is a constant from Table 1.

If we set the value of κ to 0, we actually obtain the empirical size. In addition, we consider two different covariance structures.

**Table 1.** Values of the step $s^{*}$ for different alternative simulation designs.

Dimension	$n_{1}=100,n_{2}=20$	$n_{1}=20,n_{2}=20$
κ	$3*10^{-3}$	$3*10^{-2}$

- Independent: $\Sigma_{I}=I$
- Spike eigenvalue: Denote $\Sigma_{SC}=0.3I+0.71_{p}1_{p}^{\prime }$, where **1** is a column vector with all elements being 1.

**Figure 2.**
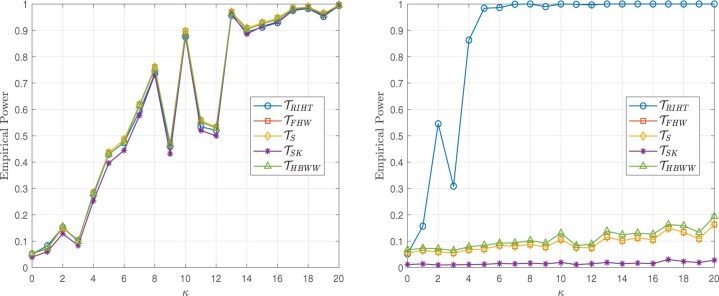
Empirical powers for the Independent setting (left), Spike eigenvalue setting (right), and sample size $\left(n_{1},n_{2})=(100,20\right)$ with the Model I.

**Figure 3.**
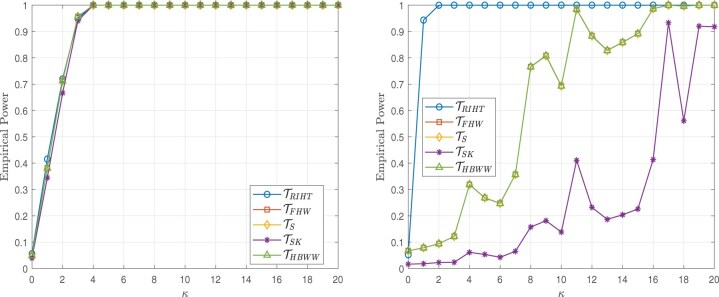
Empirical powers for the Independent setting (left), Spike eigenvalue setting (right), and sample size $\left(n_{1},n_{2})=(20,20\right)$ with the Model I.

**Figure 4.**
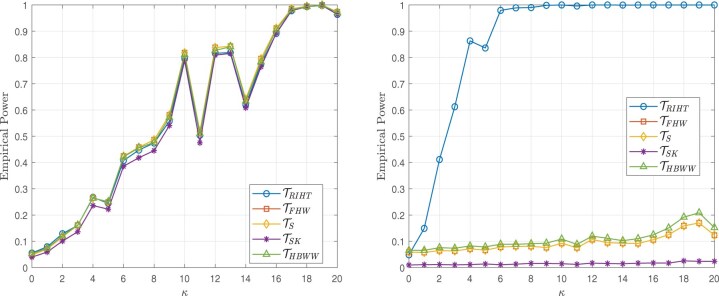
Empirical powers for the Independent setting (left), Spike eigenvalue setting (right), and sample size $\left(n_{1},n_{2})=(100,20\right)$ with the Model II.

**Figure 5.**
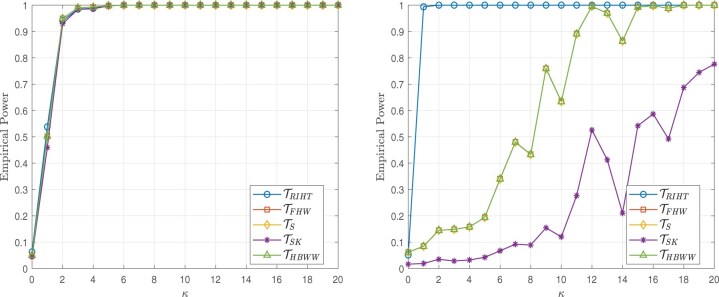
Empirical powers for the Independent setting (left), Spike eigenvalue setting (right), and sample size $\left(n_{1},n_{2})=(20,20\right)$ with the Model II.

The simulation results presented in this section are based on 5000 simulations conducted at a nominal significance level of 0.05. The results are illustrated in Figs 2–5, respectively.

From Figs 2–5, the proposed test occasionally exhibits similar power to other methods. However, in the case of spike eigenvalues, the proposed test demonstrates the highest power, outperforming the alternatives. This demonstrates that our method outperforms other models in high-dimensional scenarios where the distribution is unknown and the data is general, highlighting its robustness and effectiveness under these challenging conditions.

### 4.2 Simulation for precision matrices estimation and testing

We utilized the proposed method to simultaneously estimate the precision matrix and infer structural changes. The hyper-parameters are selected by the algorithm (Algorithm 3). In this section, to demonstrate the performance of the FGL and also the tuning method, we compare it with the AIC tuning method. We consider a two-sample precision matrix testing, i.e. G=2, and the two-sample hypothesis test problem is

(37) $$H_{0}:\Theta_{1;ij}^{0}=\Theta_{2;ij}^{0}=\Theta_{0,ij}\;vs.\;H_{1}:\Theta_{1;ij}^{0}\neq \Theta_{2;ij}^{0},$$

with $a_{1}=1$ and $a_{2}=-1$. We simulate $X_{1}$ and $X_{2}$, which are multivariate normal distributed with mean $0_{p}$, population covariance matrices $\Sigma_{g}$, and the population precision matrices $\Theta_{g}^{0}=\Sigma_{g}^{-1}$, for g=1,2. Let $D_{s}$ be a p×p adjacency matrix with s-sparsity level and its diagonal elements be zeros. Denote U be p×p matrix, whose entries $u_{ij}$ are uniformly distributed on the interval $\left(u_{\min},u_{\max}\right)$. In general, we set $u_{\min}=0.5$ and $u_{\max}=1$. Let

(38) $$\Theta_{0}=\left((D_{s}{}^{\circ}U)+{\left(D_{s}{}^{\circ}U\right)}^{\prime }\right)/2+\epsilon I_{p},$$

where “°” denotes the Hadamard product of two matrices, ϵ is a perturbation term. For the nondiagonal parts, there are 100s% entries are 1, where s=card(S)/p(p−1), and $S=\{(i,j):\Theta_{0;ij}\neq 0,i\neq j\}$. Under these configurations, we generate datasets based on different scenarios by adjusting the sample size, dimension and sparsity. Six scenarios are conducted in our studies, and the dimensions are 50 for all scenarios.

1. **Scenario I**: High sparsity, small sample size. In this case, the sparsity is set to 0.1, the sample sizes $n_{1}$ and $n_{2}$ are set to 200.
2. **Scenario II**: High sparsity, large sample size. The sparsity is the same as Scenario I, but the sample sizes is set to 500.
3. **Scenario III**: Low sparsity, small sample size. We set the sparsity to 0.3 and $n_{1}=n_{2}=200$ in this case.
4. **Scenario IV**: Low sparsity, large sample size. The sparsity is the same as Scenario III, but the sample size is set to 500.
5. **Scenario V**: Relatively dense, small sample size. We set the sparsity to 0.5 and $n_{1}=n_{2}=200$ in this case.
6. **Scenario VI**: Relatively dense, large sample size. The sparsity is the same as Scenario V, but the sample size is set to 500.

For each scenario, we generate a set of samples $X_{1}$ and $X_{2}$ 500 times based on the settings independently and randomly, and then use the proposed method tuning the hyper-parameters by Algorithm 3. Suppose the tuning hyper-parameter is ${\hat{\lambda }}_{h}$, then we use this parameter in FGL and obtain ${\hat{\Theta }}_{h}$; further, we calculate the MPDe test statistics for hypothesis (37) under null, which is

$$T_{\text{MPDe};ij}(X_{1},X_{2})=\frac{\sqrt{n}[T_{ij}-\Theta_{ij}^{0}]}{{\hat{\sigma }}_{ij}},$$

where

$$\begin{array}{c} T_{ij}:={\left({\tilde{\Theta }}_{1}-{\tilde{\Theta }}_{2}\right)}_{ij},{\tilde{\Theta }}_{1}=2{\hat{\Theta }}_{1}-{\hat{\Theta }}_{1}S_{n_{1}}{\hat{\Theta }}_{1},\\ {\tilde{\Theta }}_{2}=(2{\hat{\Theta }}_{2}-{\hat{\Theta }}_{2}S_{n_{2}}{\hat{\Theta }}_{2}),\Theta_{ij}^{0}=\Theta_{1;ij}^{0}-\Theta_{2;ij}^{0}, \end{array}$$

and

$${\hat{\sigma }}_{ij}^{2}={\tilde{\Theta }}_{1;ii}{\tilde{\Theta }}_{1;jj}+{\tilde{\Theta }}_{1;ij}^{2}+{\tilde{\Theta }}_{2;ii}{\tilde{\Theta }}_{2;jj}+{\tilde{\Theta }}_{2;ij}^{2}.$$

When we conduct the comparison, the AIC method finds the optimal tuning parameters through searching the grid. Both $\lambda_{1}$ and $\lambda_{2}$ are set ranging from 0.05 to 0.3 with step (0.3−0.05)/(30−1)=0.0034.

We present the results obtained from analyzing the simulated dataset, consisting of two parts. The first one is the results of estimation. As the precision matrix exhibits sparsity, our goal is for the estimation algorithm to preserve this sparse structure. Hence, we use the recall measure to assess how accurately our proposed method can estimate the sparse matrix. Let

$$\begin{array}{c} S_{1}^{\left(1\right)}=\{(i,j):{\tilde{\Theta }}_{1;ij}\neq 0,\Theta_{0;ij}\neq 0,i\neq j\},\\ S_{1}^{\left(2\right)}=\{(i,j):{\tilde{\Theta }}_{2;ij}\neq 0,\Theta_{0;ij}\neq 0,i\neq j\}. \end{array}$$

Under null hypothesis, ${\tilde{\Theta }}_{1;ij}$ and ${\tilde{\Theta }}_{2;ij}$ have same structure of sparsity, thus we denote $S_{1}=S_{1}^{\left(1\right)}=S_{1}^{\left(2\right)}$. Similarly, we denote

$$S_{0}=\{(i,j):{\tilde{\Theta }}_{1;ij}=0,\Theta_{0;ij}=0,i\neq j\}.$$

Then, the recall is defined by

$$R_{1}=\frac{\mathrm{card}(S_{1})}{p(p-1)s}*100\%,\;R_{0}=\frac{\mathrm{card}(S_{0})}{p(p-1)(1-s)}*100\%,$$

where s is the sparsity level. The recall reflects whether the estimation ${\tilde{\Theta }}_{h}$ covers over the real precision matrix $\Theta_{0}$ well. In other words, it means how the method can distinguish the nonzero elements with zero elements in the precision matrix correctly. As hyperparameters significantly impact the model’s performance, we use the proposed data-driven method to tune the hyperparameters and obtain the optimal ones initially. Similarly, we use the AIC to tune the hyperparameters for comparison purposes in the following experiments. Subsequently, with the optimal hyperparameters fixed, we proceed to estimate the precision matrix. Following estimation, we examine the reliability of the hypothesis testing part by simulations. If the null hypothesis is true, it holds that $\Theta_{ij}^{0}=0$. According to the limiting result of the proposed test (21), with 500 independent replications, the frequency histogram of statistic $T_{\mathrm{MPDe};ij}$ should resemble the density curve of standard normal distribution. So does the smoothed kernel density curve. It reflects whether $T_{\mathrm{MPDe};ij}$ test is reasonable and valid. Hence, we compare the kernel density curve with the simulated data to the standard normal distribution density curve at last.

#### 4.2.1 Accuracy and computational time

After running the algorithm to estimate the precision matrix of simulated data 500 times across four scenarios: **Scenario I**, **Scenario II**, **Scenario III**, **Scenario IV**, we calculate the average *Accuracy*, $R_{1}$ and $R_{0}$ indices, summarized in Table 2.

**Table 2.** Evaluating the performance of estimating precision matrix by two tuning methods across four scenarios with G=2.

	Scenario I		Scenario II		Scenario III		Scenario IV	
	DD	AIC	DD	AIC	DD	AIC	DD	AIC
Accuracy	94.75%	88.61%	93.88%	87.46%	74.69%	75.59%	72.66%	69.60%
$R_{1}$	69.00%	49.47%	70.36%	47.76%	64.43%	58.54%	64.68%	51.88%
$R_{0}$	99.49%	99.99%	97.44%	100.00%	77.39%	87.09%	74.45%	98.43%
Accuracy	94.74%	88.58%	93.88%	87.40%	74.69%	75.60%	72.66%	69.63%
$R_{1}$	68.96%	49.40%	70.36%	47.66%	64.43%	58.56%	64.68%	51.91%
$R_{0}$	99.49%	99.99%	97.44%	100.00%	77.39%	87.09%	74.45%	98.40%

**Figure 6.**
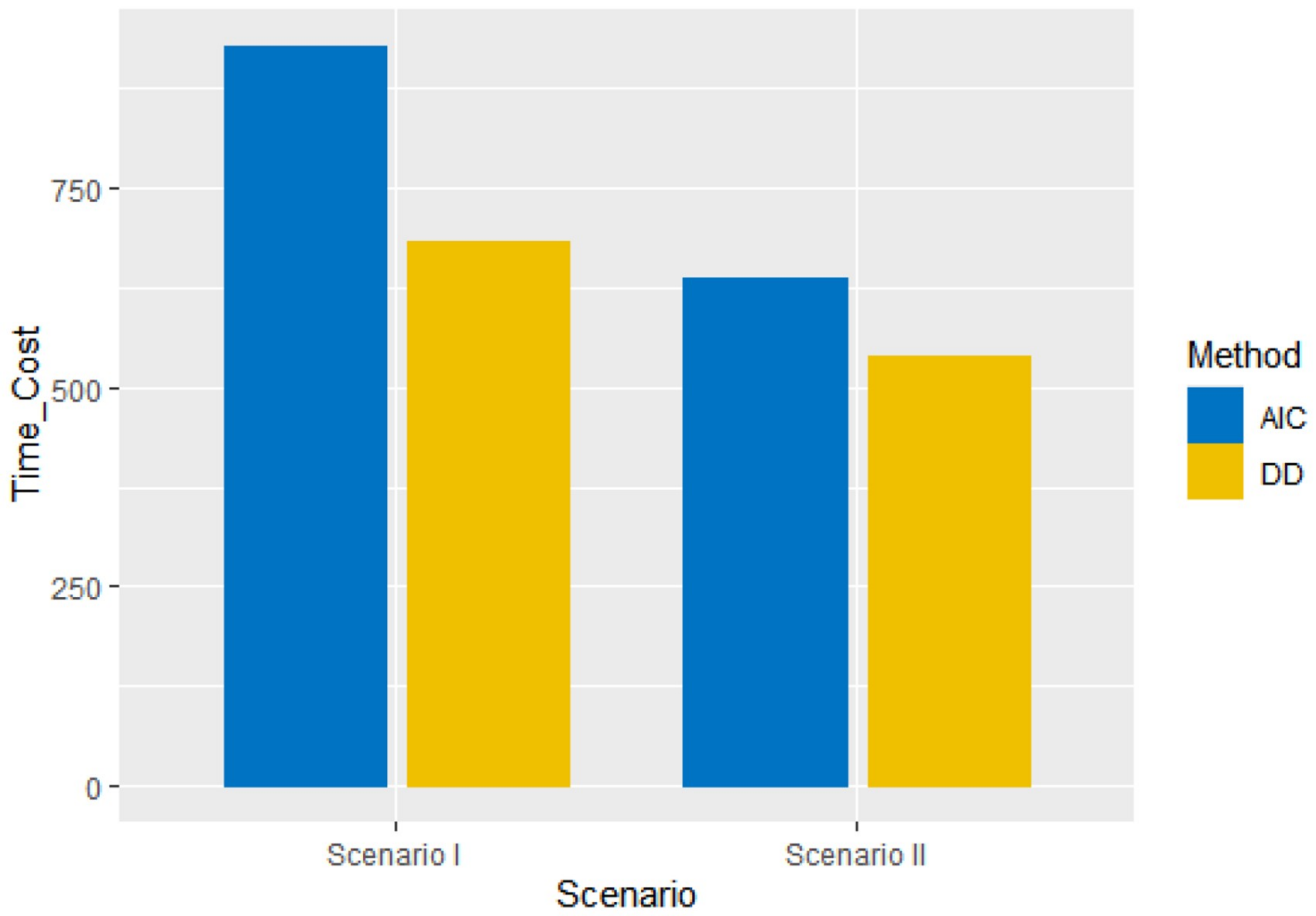
The time-consuming of hyperparameter tuning using DD and AIC with simulated data **(Scenario I–II).**

Results in Table 2 indicate the proposed method outperforms AIC. It can estimate and infer the precision matrix when the sparsity is larger. The last part presents the computational time consumed. In addition, we compare the tuning times of the data-driven method and the AIC method. We calculate the tuning time by the same sample generated from **Scenario I–II**. The standard of measure of time is second, and the result is present in Table 2 and Fig. 6, demonstrate that the data-driven method can save the calculation time, and the estimation based on the data-driven method is more accurate than AIC method.

#### 4.2.2 Asymptotic analysis

**Figure 7.**
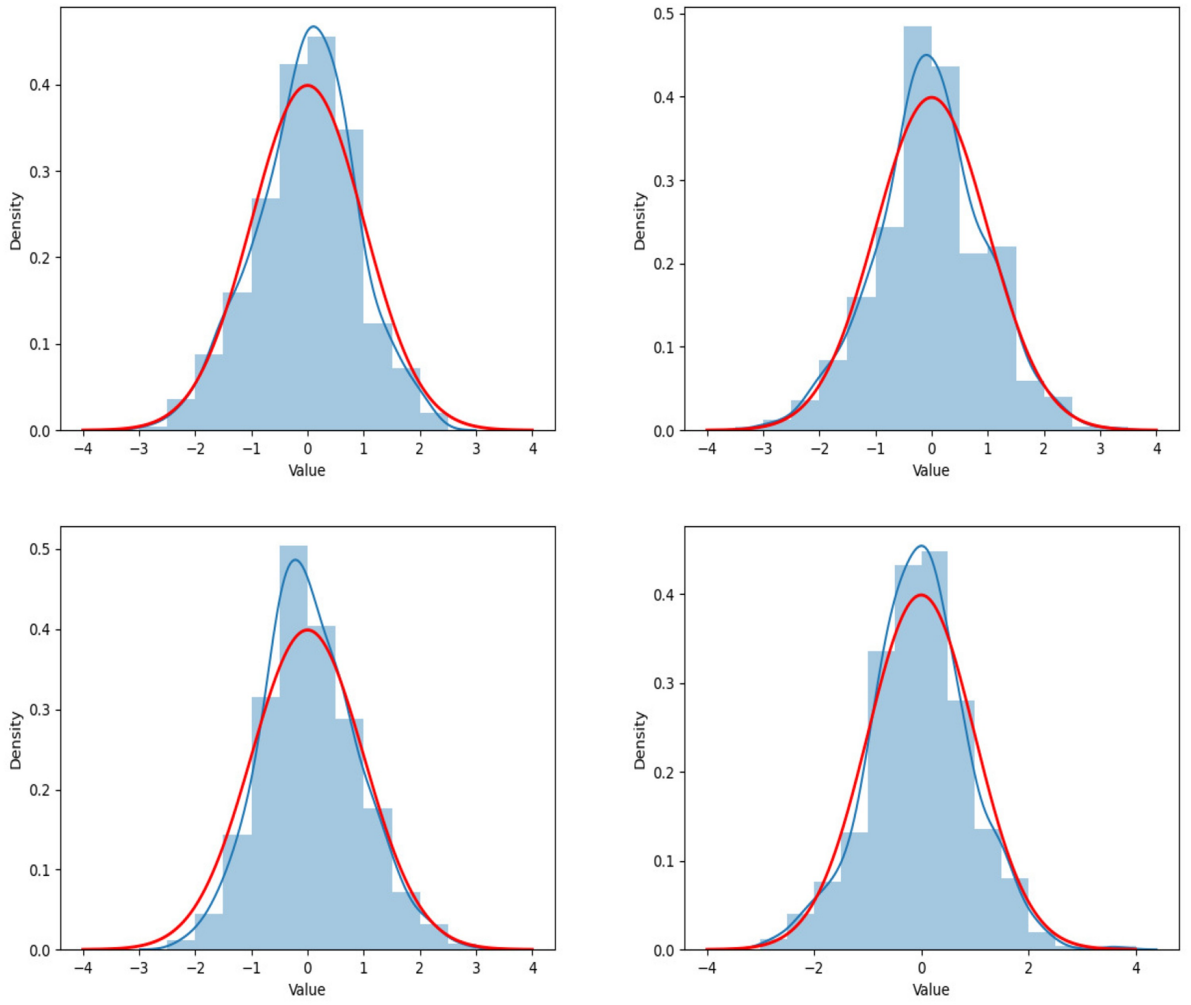
The figure shows the fluctuation of the $T_{\text{MPDe};ij}$, its kernel density curve (blue line) and standard normal distribution density curve (red line) at different locations (i,j) under **Scenarios V** setting. The values of $T_{\text{MPDe};ij}$ in the graphs are constructed by the estimated precision matrices whose parameters are tuning by the DD method. The locations of the four sub-graphs, arranged from the top left to the bottom right, are (1,3, 2,2, 2,4), and (6,6), which coincide with those listed in Table 3.

**Figure 8.**
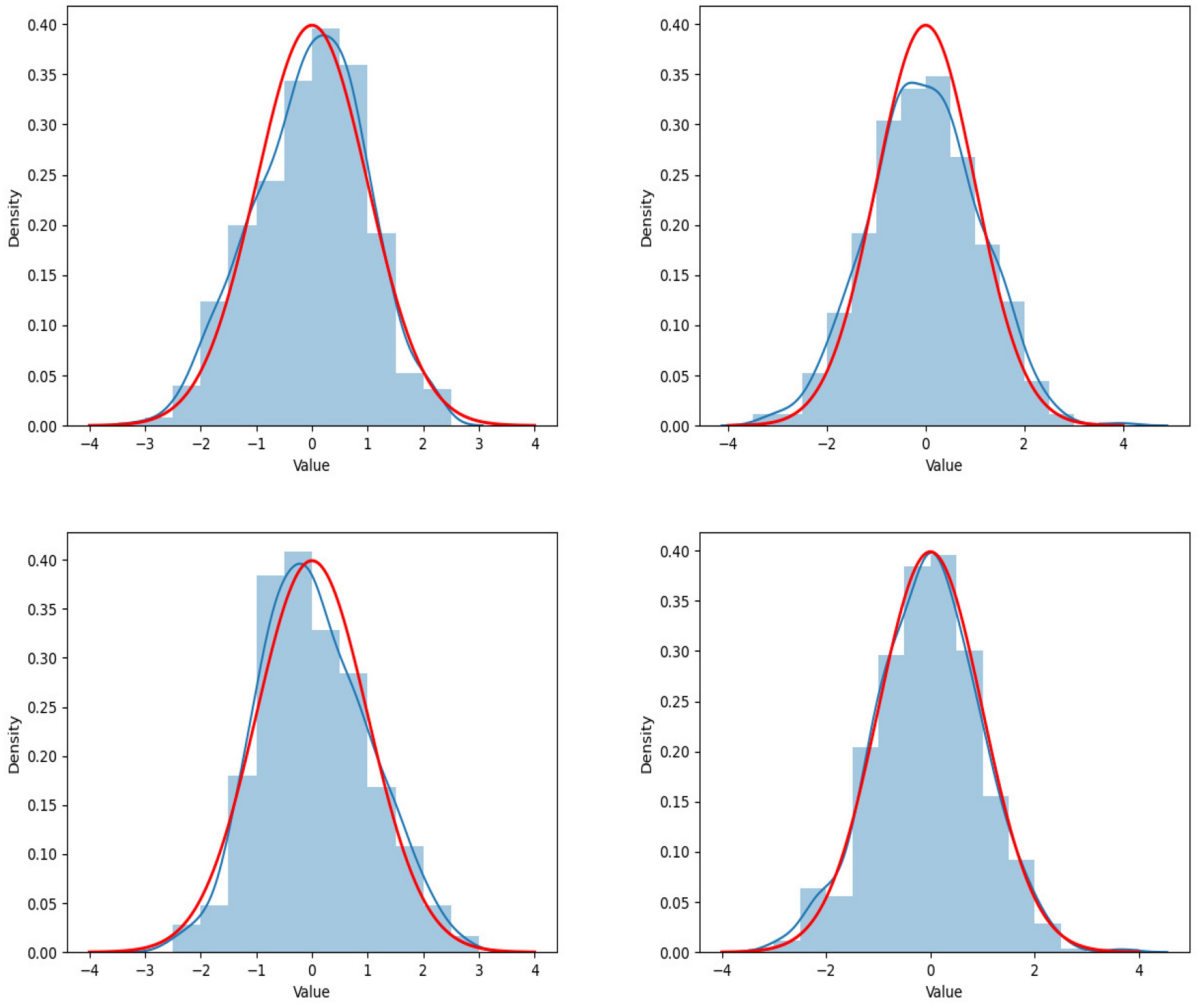
The figure shows the fluctuation of the $T_{\text{MPDe};ij}$, its kernel density curve (blue line) and standard normal distribution density curve (red line) at different locations (i,j) under **Scenarios V** setting. The values of $T_{\text{MPDe};ij}$ in the graphs are constructed by the estimated precision matrices whose parameters are tuning by the AIC method. The locations of the four sub-graphs, arranged from the top left to the bottom right, are (1,3, 2,2, 2,4), and (6,6), which coincide with those listed in Table 3.

**Figure 9.**
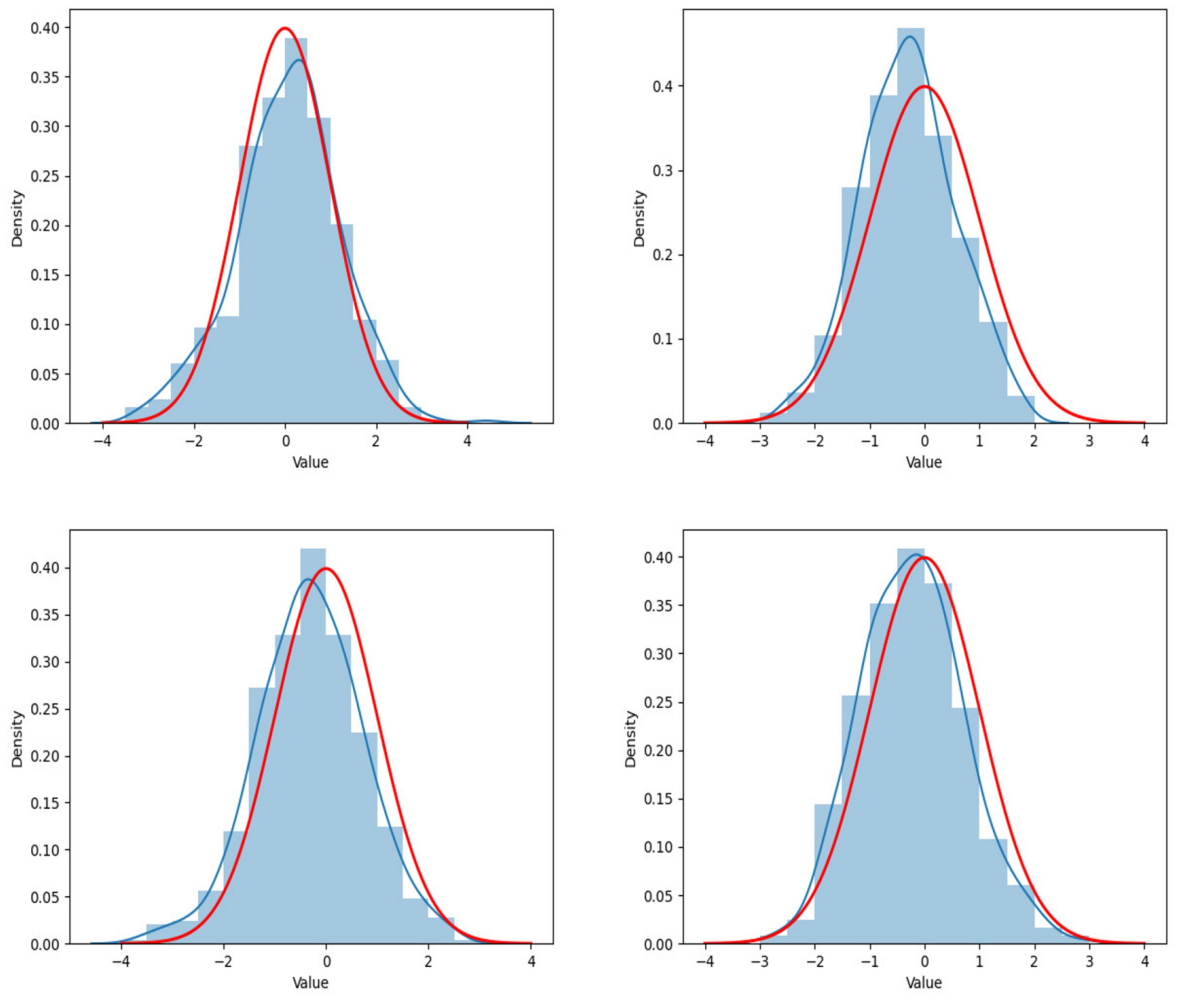
The figure shows the fluctuation of the $T_{\text{MPDe};ij}$, its kernel density curve (blue line) and standard normal distribution density curve (red line) at different locations (i,j) under **Scenarios VI** setting. The values of $T_{\text{MPDe};ij}$ in the graphs are constructed by the estimated precision matrices whose parameters are tuning by the DD method. The locations of the four sub-graphs, arranged from the top left to the bottom right, are (1,9, 3,10, 4,10) and (15,12), which coincide with those listed in Table 3.

**Figure 10.**
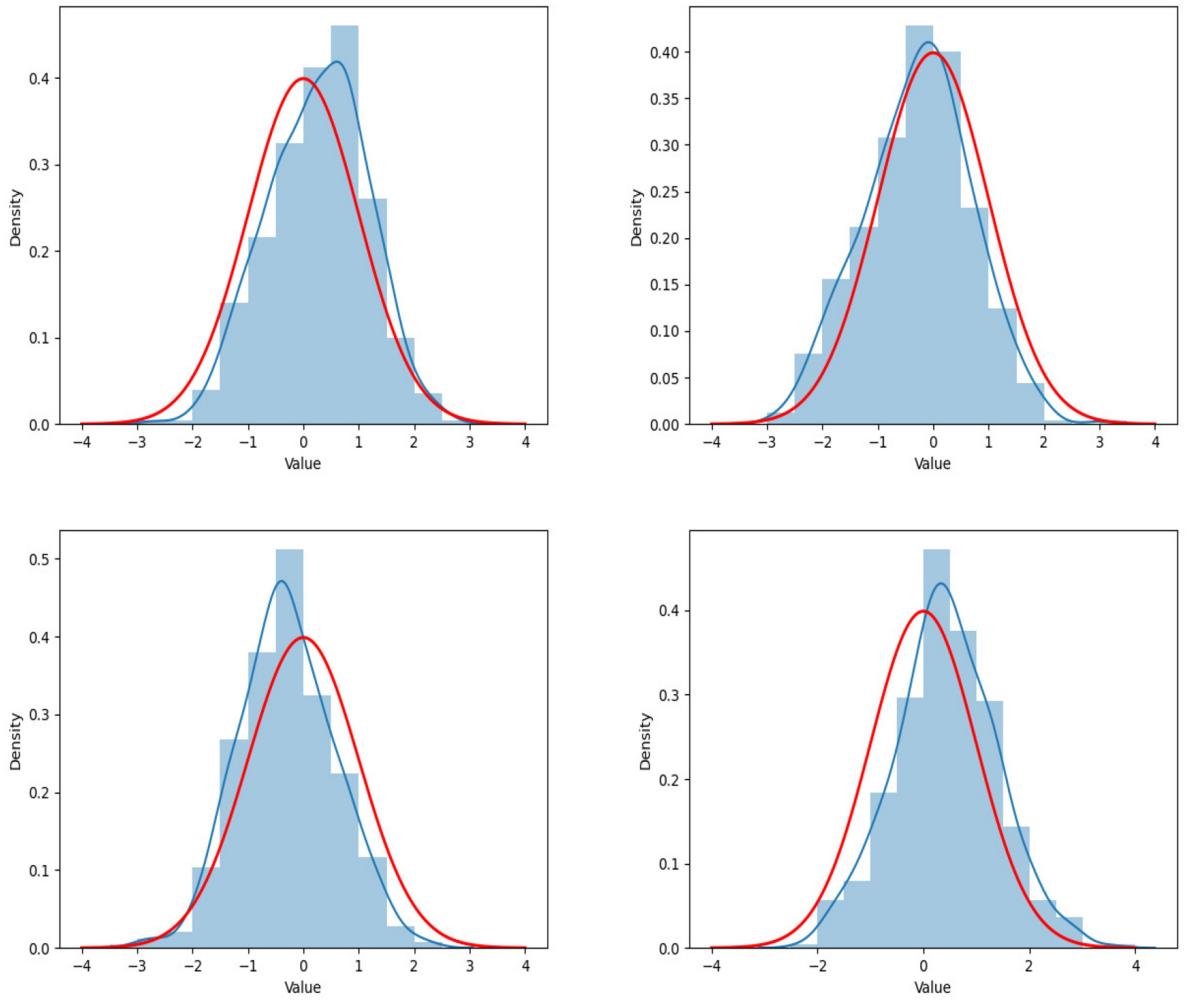
The figure shows the fluctuation of the $T_{\text{MPDe};ij}$, its kernel density curve (blue line) and standard normal distribution density curve (red line) at different locations (i,j) under **Scenarios VI** setting. The values of $T_{\text{MPDe};ij}$ in the graphs are constructed by the estimated precision matrices whose parameters are tuning by the AIC method. The locations of the four sub-graphs, arranged from the top left to the bottom right, are (1,9, 3,10, 4,10) and (15,12), which coincide with those listed in Table 3.

In this subsection, we show the kernel density curve to demonstrate whether the empirical distribution of the statistic can be well fitted by its limiting distribution. The concerned hypothesis problem is testing the linear combination of entries on the same location of several different precision matrices. Thus, the statistic $T_{\mathrm{MPDe};ij}$ is also estimated by a function of entries on the same location of estimated precision matrices. For different locations, we execute a two-sample testing problem in (37), where $\Theta_{ij}^{0}$ is generated by (38), and we produce the testing procedure with 500 independent replications for two scenarios: **Scenario V** and **Scenario VI**, i.e. we conduct the simulation studies within different sample size. The reason why we choose a relatively dense case in this subsection is that we can present the results at more nonzero locations. We record the locations at Table 3. The renderings of fitness are shown in Figs 7–10, and it fully shows that the proposed test method is feasible. The testing process is carried out under the condition that the null hypothesis is true. Therefore, according to the theorem, the test statistic consisted of estimations of the precision matrices has an asymptotic standard normal properties. It can be seen from Figs 7 to 10 that the empirical distribution of the proposed test statistics fits with the standard normal distribution well under all scenarios.

**Table 3.** The locations of (i,j) within two scenarios: **Scenarios V**, and **Scenarios VI.**

Scenarios V	(1,3)	(2,2)	(2,4)	(6,6)
Scenarios VI	(1,9)	(3,10)	(4,10)	(15,12)

## 5 Applications

### 5.1 Experiment settings

The perception of facial features plays a vital role in social cognition (Krawczyk 2017). The information conveyed by a face enables individuals to discern identity, interpret thoughts and emotions, anticipate actions, recognize feelings, foster connections, and communicate effectively through body language. When audiences view different face images, their emotions may vary in the body and brain states that originate from external stimuli that influence cognitive processing (Damasio *et al.* 1994, Bechara and Damasio 2005). It plays a vital role in daily life, influencing not only human interaction but also decision-making processes. For instance, when businesses use facial images to represent products, services, or advertisements, related analyses can assess the effectiveness of these images in fostering emotional connections between customers and the offerings. This understanding helps businesses develop more effective marketing strategies that leverage the use of facial images (Khondakar *et al.* 2024). Additionally, the dynamics of emotions in daily life have been widely studied in clinical and well-being contexts (Damasio and Carvalho 2013).

To validate the proposed EEG data analysis framework, we designed four distinct scenarios to collect separate sets of EEG data related to facial recognition. In each scenario, EEG data were independently collected from 27 healthy student participants, resulting in each dataset containing 27 samples across 63 channels. The participants had a mean age of 24.13 years (SD=3), with 14 females, accounting for 30% of the total. During data collection, participants first viewed 64 images (240 × 240 pixels, displayed on a black background), including 32 Asian faces and 32 White faces. Then, in the subsequent response phase, participants were required either to categorize the viewed faces belonging to the Asian or White, or to determine their memorization of Asian and White faces. Considering the relationship between the racial of viewing faces and response types, four types of experiment settings were developed, and four corresponding datasets were collected, as illustrated in Table 4.

**Table 4.** The settings of four distinct Experiment settings.

Datasets	Task type	Experiment settings	Sample size	Time range	Electrodes
I	Categorization	Participants viewed random racial faces, then categorized whether the present face is Asian or not.	27	−200 to 800 ms	63
II	Categorization	Participants viewed random racial faces, then categorized whether the present face is White or not.	27	−200 to 800 ms	63
III	Memory Test	Participants viewed random racial faces, then tested for memorization of Asian faces.	27	−200 to 800 ms	63
IV	Memory Test	Participants viewed random racial faces, then tested for memorization of White faces.	27	−200 to 800 ms	63

### 5.2 Data collection and processing

**Figure 11.**
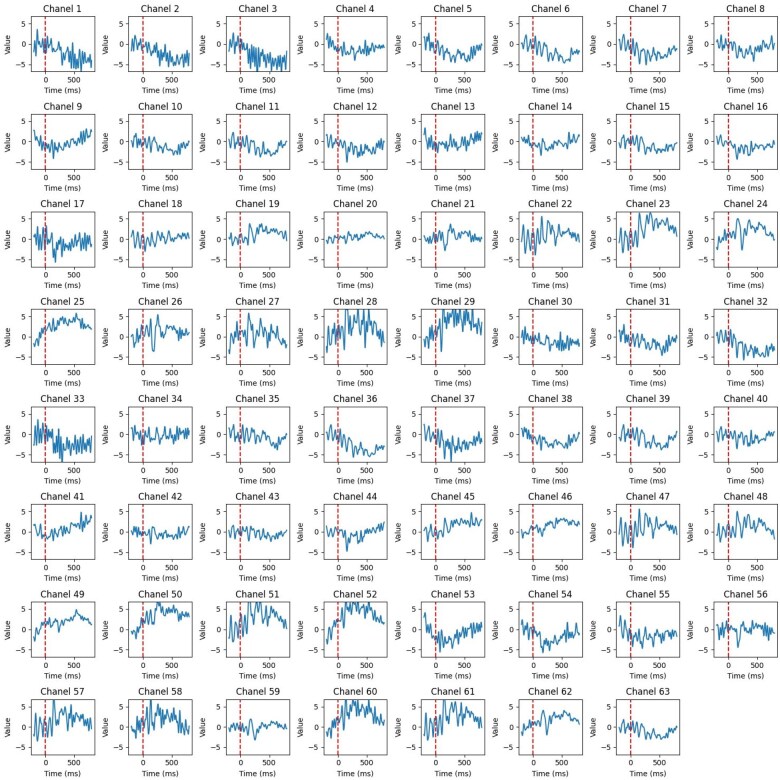
An example of EEG signal data.

To ensure consistent experimental conditions and the quality of EEG data, all participants were seated in a stationary chair in a dimly lit room, positioned approximately 60 cm from the screen. EEG data were collected using a flexible cap equipped with 64 sensors (Ag/AgCl electrodes) arranged according to the standardized 10–20 system for brain activity recording. The data were referenced to a central electrode and recorded at a sampling rate of 500 Hz using an AsaLab amplifier (www.ant-neuro.com). After recording, offline EEG signal data was re-referenced to mastoid electrodes and band-pass filtered (0.1–30 Hz) using EEGLab toolbox, 63 electrodes were left and entered the further processing. The eye blink artifacts of the signal were corrected. Nonbrain signal components were distinguished and rejected using an independent component analysis (ICA EEGLAB toolbox with ICLabel) (Delorme and Makeig 2004, Wang and Samworth 2018), and remaining amplitudes exceeding ± 100 mV were also rejected to ensure signal quality. The EEG data was epoched from −200 to 800 ms relative to the racial face onset, and −200 ms was the baseline. Finally, each experiment independently collected 27 samples from 27 participants. Each sample consisted of a multivariate time series containing 63 channels, recorded by corresponding electrodes over a time range from −200 to 800 ms, shown in Table 4. To illustrate the EEG data, one individual’s is presented, highlighting the signals recorded from 63 electrodes over a time range of −200 to 800 ms, shown in Fig. 11.

### 5.3 Performing the RIHT test to verify changes in mean vectors

To evaluate the effectiveness of the proposed method, the testing approach is applied to verify changes in the mean vectors of the collected EEG data. Reviewing the proposed RIHT test method, it computes the statistical difference between the mean vectors of brain electrical activity signals before and after the stimuli, enabling a decision on whether the stimuli have a significant impact on EEG changes. If the statistical value is larger than the significant level, it indicates that the stimuli have a significant effect on EEG changes.

Specifically, let the EEG signal data for an individual in an experiment be represented as a matrix**X** where the data consists of 63 channels (dimensions) and spans 500 time points, covering both the pre- and post-stimulus periods. P1 and N170 are crucial components for cognitive processing, as validated in several studies summarized in the literature review, specifically, reflecting early visual processing, with the P1 typically associated with initial attention allocation and sensory processing, while the N170 is often linked to face and object recognition (Ahumada-Mendez *et al.* 2022). Therefore, we divided each EEG dataset was into three epochs: (i) Baseline interval: From the beginning to the stimulus point, including 100 sample points. (ii) P1 interval: from 120–160 ms, including 20 sample points. (iii) N170 interval: from the point at 200–220 ms, including 20 sample points. Through assessing the performance of the RIHT of those 3 epochs, our goal is to determine whether the RIHT can effectively detect the impact of the stimuli. If it proves successful, this would indicate that the method can identify how stimuli influence changes in EEG signals, suggesting either a state transition due to stimulation or an inherent shift in brain activity. Therefore, we conduct tests on the mean vectors of these intervals, as well as on the mean vectors of any two intervals, specifically between the Baseline interval and the P1 interval, between the P1 interval and the N170 interval, and between the Baseline interval and the N170 interval.

All results are based on the analysis of data collected from four experiments. Each experiment involved 27 individuals, so changes in the mean vector were tested individually for each of the 27 participants. The results in Table 5 can be interpreted as a test for differences in mean vectors across multiple populations. Correspondingly, the results in Tables 6–8 represent tests for changes between two adjacent intervals. Note: Tables 5–8 present the values of the RIHT test statistic with the unit $10^{3}$. By comparing the RIHT test statistic with the threshold 1.96 from the normal distribution, it can be determined whether the result is statistically significant.

**Table 5.** The RIHT test for verifying changes in the mean vector across three intervals: baseline State, P1 State, and N170 State.

Individual	Experiment			
	I	II	III	IV
No.1	0.2543	0.3050	0.3220	0.3447
No.2	0.7122	0.5097	0.6535	0.7087
No.3	0.3276	0.3319	0.4873	0.3248
No.4	0.4105	0.6152	0.5389	0.7650
No.5	0.6241	0.5730	0.4898	0.5435
No.6	0.6001	0.7730	0.6766	0.7848
No.7	0.5771	0.6150	0.3780	0.3658
No.8	0.5013	0.4478	1.0657	1.2476
No.9	0.4716	0.3261	0.9047	0.6467
No.10	0.5151	0.6227	1.3905	0.7253
No.11	0.4024	0.8304	0.9502	1.0322
No.12	0.2033	0.2095	0.1214	0.1309
No.13	0.4947	0.7256	0.5369	0.5445
No.14	1.2278	0.7237	0.6866	0.8085
No.15	0.3573	0.4170	0.8691	0.7669
No.16	0.3818	0.6584	0.4496	0.6613
No.17	0.6722	0.9714	1.1653	1.2567
No.18	0.6816	0.9106	1.1325	1.2543
No.19	0.8353	1.2256	0.9552	0.9904
No.20	0.5582	0.5126	0.6765	0.5776
No.21	0.5124	0.5998	0.6134	0.9307
No.22	0.4432	0.4821	0.7269	1.2361
No.23	0.5256	0.8141	0.6623	0.6651
No.24	0.5358	0.5472	0.5348	0.4325
No.25	0.6546	0.5216	0.7665	1.4300
No.26	0.4676	0.3855	0.8536	1.0820
No.27	0.3967	0.4009	0.2879	0.5339

**Table 6.** The RIHT test for verifying changes in the mean vector between two intervals: baseline state and P1 state.

Individual	Experiment			
	I	II	III	IV
No.1	0.2072	0.2135	0.2511	0.2177
No.2	0.7677	0.7001	0.6033	0.5230
No.3	0.4092	0.4529	0.5300	0.3812
No.4	0.3629	0.6507	0.2775	0.3433
No.5	0.4764	0.1263	0.2139	0.2852
No.6	0.6094	0.9426	1.1724	0.7859
No.7	0.2945	0.2549	0.2032	0.1436
No.8	0.2408	0.1627	0.2282	0.2564
No.9	0.4306	0.2323	0.9228	0.6099
No.10	0.1700	0.3045	0.4121	0.3280
No.11	0.3007	0.5673	0.3427	0.3325
No.12	0.1511	0.1383	0.0847	0.1106
No.13	0.2882	0.4181	0.2333	0.4220
No.14	0.9840	0.4476	0.4889	0.4819
No.15	0.1596	0.2399	0.0609	0.1155
No.16	0.2497	0.4462	0.1321	0.2080
No.17	0.5293	0.3255	0.1922	0.2819
No.18	0.6176	0.3264	0.2008	0.2842
No.19	0.1093	0.1334	0.1661	0.1865
No.20	0.4353	0.4918	0.2441	0.2346
No.21	0.3507	0.4022	0.4334	0.5101
No.22	0.0847	0.0978	0.0821	0.0922
No.23	0.4412	0.8379	0.7437	0.6563
No.24	0.6620	0.6545	0.7062	0.4696
No.25	0.0712	0.0755	0.0818	0.1505
No.26	0.3019	0.3312	0.3052	0.2802
No.27	0.1152	0.1262	0.1795	0.3748

**Table 7.** The RIHT test for verifying changes in the mean vector between two intervals: baseline state and N170 state.

Individual	Experiment			
	I	II	III	IV
No.1	0.1958	0.2483	0.3208	0.2799
No.2	0.4879	0.2509	0.5466	0.7708
No.3	0.1492	0.2694	0.2533	0.2768
No.4	0.3183	0.3055	0.4547	0.7197
No.5	0.4353	0.8431	0.6645	0.4936
No.6	0.3381	0.2476	0.2448	0.4519
No.7	0.6474	0.5802	0.3560	0.3561
No.8	0.5949	0.4777	1.6236	1.6720
No.9	0.2892	0.2597	0.5447	0.4018
No.10	0.6578	0.6491	1.7970	0.9207
No.11	0.2686	0.5660	1.1309	1.8550
No.12	0.1573	0.1785	0.1144	0.0862
No.13	0.4756	0.6555	0.5176	0.3981
No.14	0.9775	0.6100	0.5268	0.5619
No.15	0.4152	0.4716	1.5297	1.4335
No.16	0.6119	0.6197	1.1084	1.1973
No.17	0.6384	1.6339	3.2480	2.2505
No.18	0.6670	1.5540	2.6125	2.1335
No.19	1.2997	1.6575	1.3720	1.1925
No.20	0.3732	0.3240	0.8717	0.6563
No.21	0.4604	0.9282	0.4826	0.9172
No.22	0.5845	0.6454	1.0325	1.7971
No.23	0.2908	0.4295	0.2883	0.4572
No.24	0.4389	0.4424	0.4478	0.3769
No.25	0.9831	0.8044	2.2115	1.9910
No.26	0.3910	0.2201	1.4260	1.3837
No.27	0.7747	0.8002	0.3240	0.5592

**Table 8.** The RIHT test for verifying changes in the mean vector between two intervals: P1 state and N170 state.

Individual	Experiment			
	I	II	III	IV
No.1	0.1284	0.3955	0.1863	0.3298
No.2	0.9658	0.7727	0.4154	0.3623
No.3	0.2192	0.1302	0.2057	0.1524
No.4	0.4024	0.6550	1.0947	0.7911
No.5	0.7953	0.4603	0.4778	0.3719
No.6	0.2479	0.2602	0.2039	0.2678
No.7	0.5797	0.9122	0.4483	0.3919
No.8	0.5210	0.6470	0.7627	0.7053
No.9	0.3676	0.1500	0.3458	0.2324
No.10	0.3913	0.4276	0.6820	0.3394
No.11	0.8274	1.3021	0.8599	0.5638
No.12	0.0649	0.0547	0.0707	0.0605
No.13	0.3281	0.4091	0.5822	0.3503
No.14	0.6677	0.7594	0.4966	0.6682
No.15	0.3760	0.4334	0.6551	0.4110
No.16	0.1725	0.3804	0.2379	0.3979
No.17	0.5623	0.5841	0.3859	0.6033
No.18	0.5544	0.6042	0.3733	0.6242
No.19	0.6655	0.9932	0.6282	0.7313
No.20	0.4124	0.2434	0.3262	0.3292
No.21	0.6118	0.1693	0.2502	0.3203
No.22	0.3992	0.8501	0.7633	0.7465
No.23	0.3344	0.2657	0.4801	0.1648
No.24	0.3297	0.1941	0.1553	0.2813
No.25	0.4134	0.3173	0.3763	0.9289
No.26	0.2549	0.2607	0.4906	0.8980
No.27	0.3586	0.3684	0.2976	0.5018

The results in Table 5 show that the mean vectors across the three intervals are significantly different, as the RIHT statistic values exceed the threshold of 1.96. This indicates that the stimuli have a substantial impact on overall EEG signal changes for each individual across the four experiments. Similarly, the results in Tables 6–8 also demonstrate that the mean vectors between any two intervals are significantly different. This further confirms that the tested intervals represent distinct states. All these results further illustrate the efficiency and potential applications of the RIHT test, whether for comparing mean vectors across multiple populations or between two populations.

In addition, the average of 27 individuals was calculated at each time point for each channel in each experiment to eliminate individual differences. Based on these averaged EEG data, changes in the mean vectors across multiple intervals or between two intervals are compared through the RIHT statistic. All results are presented in Table 9 with the unit $10^{3}$. These results further illustrate that the mean vectors of these intervals are different, indicating that the stimuli influence the EEG signals, leading to significant observable changes.

**Table 9.** The RIHT test for verifying changes in the mean vector based on the average EEG data.

States comparison	Experiment			
	I	II	III	IV
Baseline/P1/N170 (3 States)	0.6654	0.7640	1.3575	1.4668
Baseline/P1 (2 States)	0.2305	0.3539	0.5778	0.5536
Baseline/N170 (2 States)	0.4958	0.6019	0.5826	0.6541
P1/N170 (2 States)	0.7298	0.7673	1.8860	2.0996

**Figure 12.**
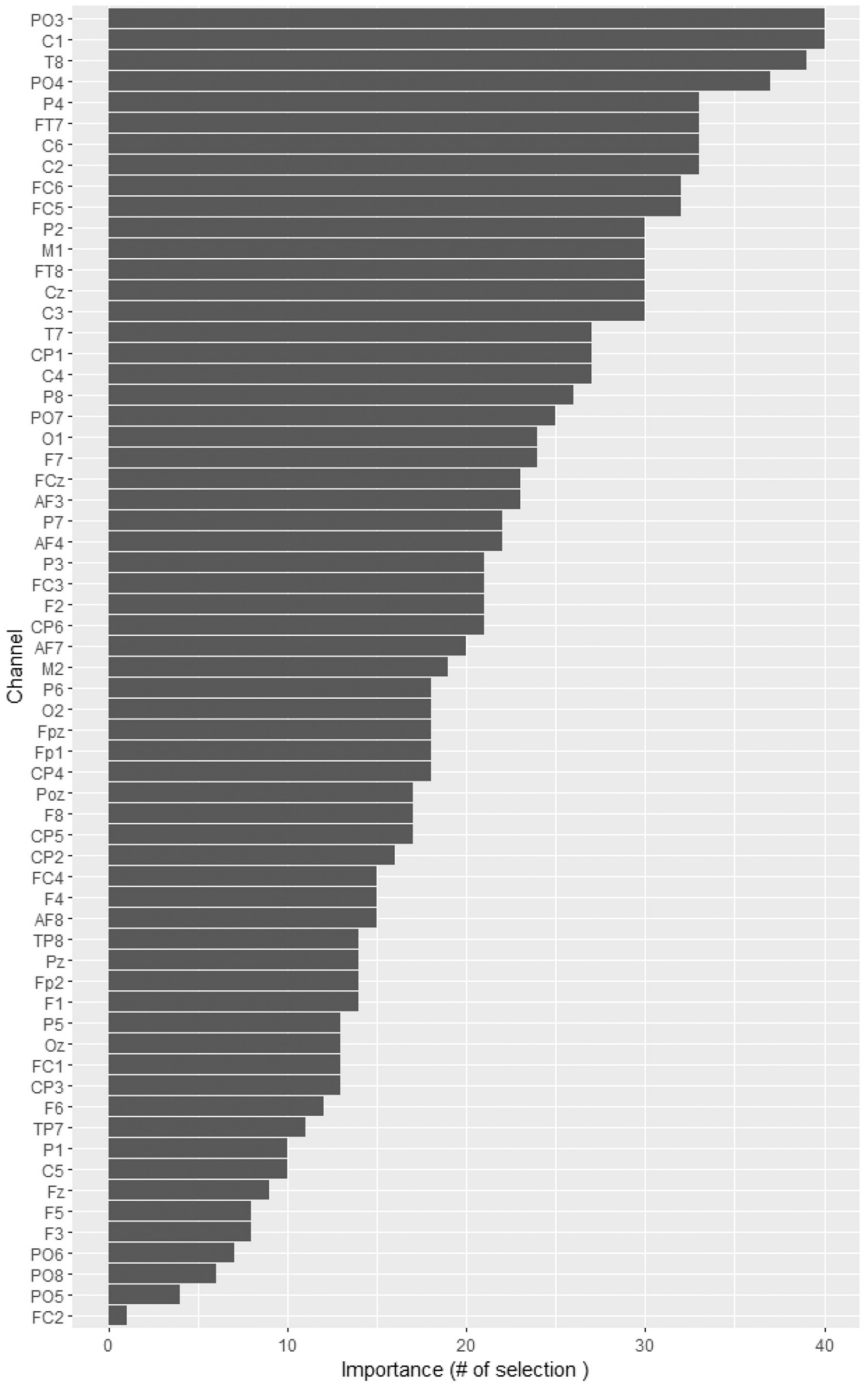
The number with which the channels are selected by the Lasso regression across all experiments.

Although hypothesis testing can determine whether differences exist in brain electrical states, it does not identify the important channels contributing to these differences. Therefore, lasso regression is applied to select and filter important channels. Lasso regression is applied to analyze the data of each individual. The dependent variable Y is defined as a binary variable, where 0 represents the value before stimulation and 1 represents the value after stimulation. The independent variables are the 63 EEG channels. For each individual, lasso regression is performed using Y as the dependent variable and X (the 63 channels) as the independent variables. The model selects the most relevant channels that best explain Y, identifying the important channels that contribute to changes in brain electrical activity following stimulation. After performing lasso regression on 4×27 samples, the frequency of channel selection is obtained and presented in Fig. 12. The height of each bar represents the frequency with which a channel was selected across individuals—the higher the bar, the more important the channel, while lower bars indicate less significance. This visualization effectively highlights the most significant channels contributing to EEG signal changes. The results from the figures indicate that the most important channels are: PO3, C1, T8, PO4, P4, and so on. The P4 channel is located in the occipital-parietal region of the brain, while the PO3 and PO4 channels are located in the parietal and occipital lobes, respectively. These brain regions play a critical role in human cognitive abilities (Haxby *et al.* 2002, Jiang *et al.* 2025). It further indicates that the high-dimensional tool can assist us in selecting the important channels.

### 5.4 Performing the MPDe to verify changes in precision matrices

In this part, we aim to estimate the conditional dependency of channels and also test which dependencies between two channes are significant. In the context of EEG signals, the precision matrix provides information about how different channels are conditional dependent on each other. A sparse precision matrix indicates that only specific channels are directly related, while a nonsparse matrix suggests more widespread dependencies among channels. Therefore, we use the joint Gaussian graphical model to estimate the sparse precision matrices of EEG signals before or after stimulation and infer the equality of them. The estimation of the precision matrix is crucial for understanding the complex relationships of channels within EEG data. Then, we obtain the significant change between EEG signals before and after stimulation through statistical testing, and construct the networks of channels of each individual to describe the dependency of EEG channels. Specifically, for each individual, we calculate the covariance matrices based on the baseline EEG signals before the stimulus, and also that based on the ERps EEG signals. We estimate the corresponding precision matrices by Algorithm 2, and also test their equality of them. The test is indeed inferring whether the conditional correlation between channels changes before and after the stimulus. The concerned hypothesis testing problem can be described as

$$H_{0}:\Theta_{1;ij}^{0}=\Theta_{2;ij}^{0}\;vs.\;H_{1}:\Theta_{1;ij}^{0}\neq \Theta_{2;ij}^{0},$$

which is exactly the hypothesis (17) with $a_{1}=1,a_{2}=-1$, and G=2, where $\Theta_{g;ij}^{0}$ is the (i,j)-entry of matrix $\Theta_{g}^{0}$ for g=1,2.

**Figure 13.**
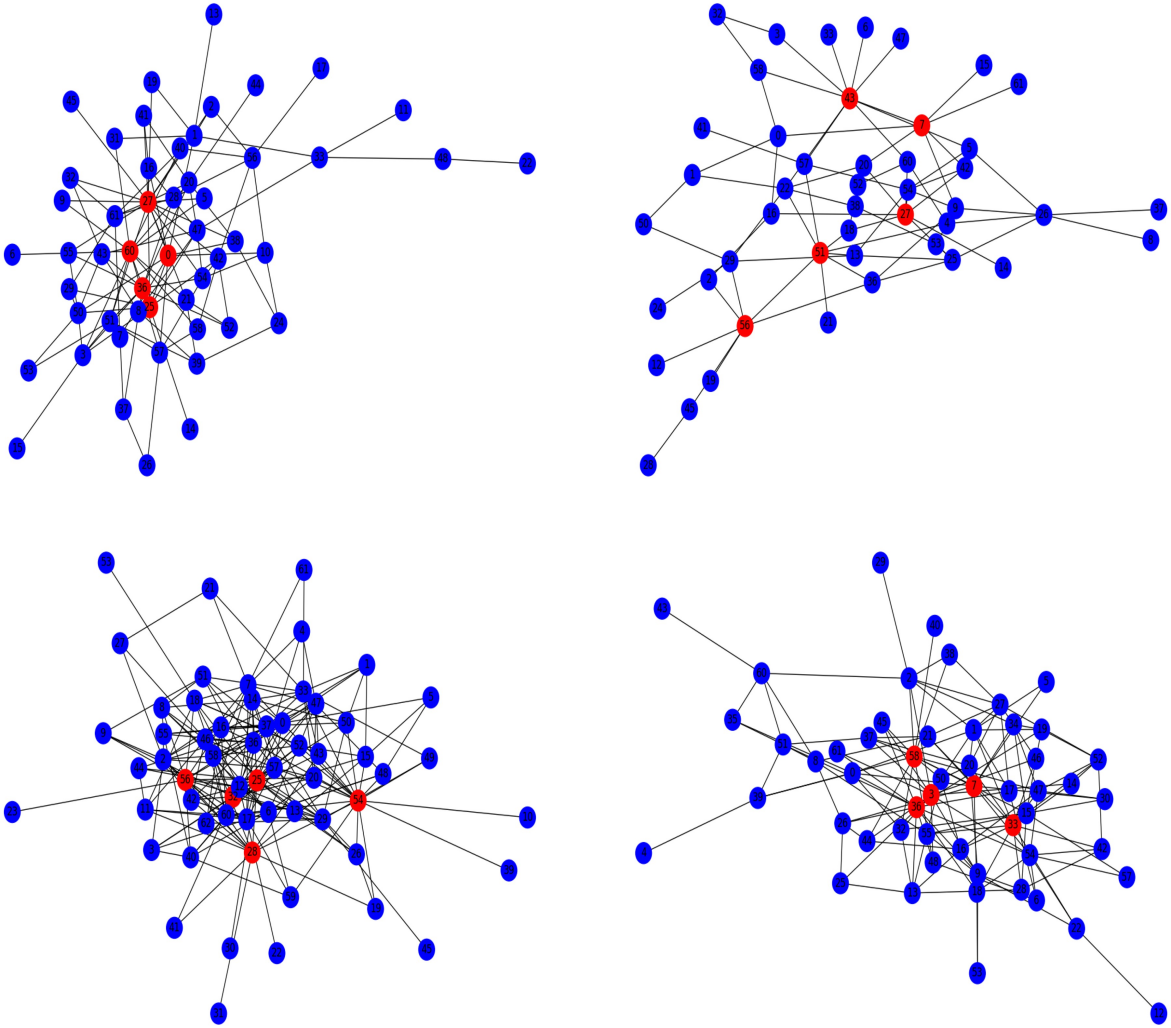
The four graphs depict the structural changes observed in individual No. 5 across four experiments. Note: Nodes with the top five highest degree centrality values are highlighted in red.

After estimating and testing, we obtain two p×p precision matrices and a p×p  p-values matrix indicating whether the conditional correlation of two channels changes before and after stimulus. If the *P*-value < 0.01, the conditional correlation of the two channels has changed significantly; otherwise, it has not changed. We only retain the significant changes and use the networks to show the dependency of channels before and after stimulus according to a precision matrix. Taking individual No. 5 as an example, the networks are shown in Fig. 13. Additionally, we assess the degree of centrality of each node for all individuals across four experiments. Nodes with the top five highest degree centrality values are highlighted in red.

**Figure 14.**
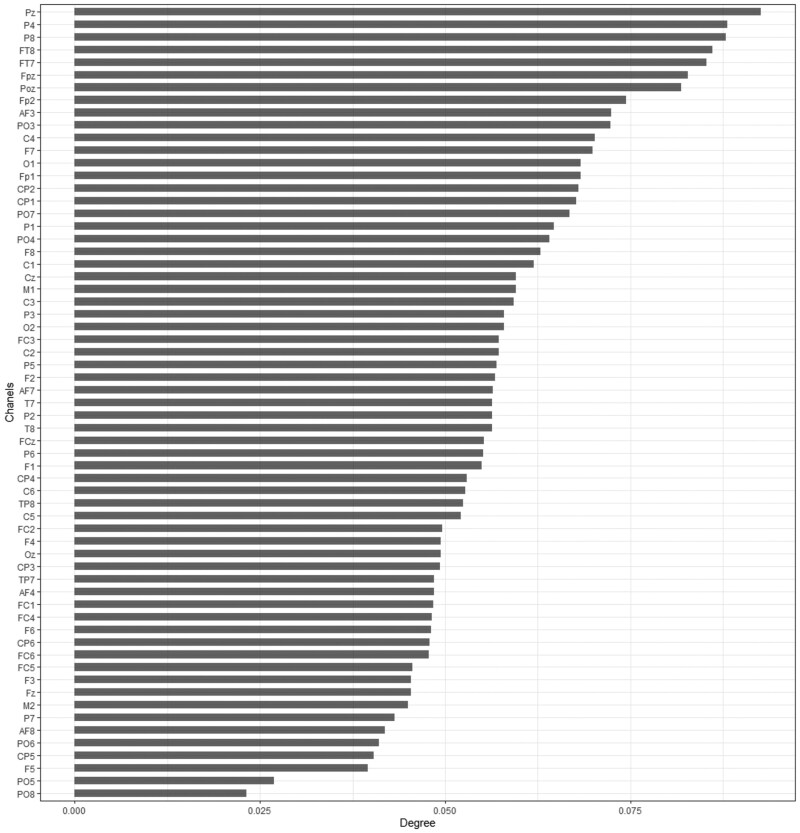
Average degree of all channels.

After conducting the connectivity analysis, we found that channels with the highest average degree in Fig. 14, including the top five channels (Pz, P4, P8, FT8, and FT7), originate from two areas associated with the visual processing of faces. This pattern reflects the neural processing of faces as identified by Bentin *et al.* (1996), referred to as the N170 component (Bentin *et al.* 1996, Rollins *et al.* 2020, Wang *et al.* 2020a). In previous studies, psychologists have faced challenges in selecting focused channels from a multitude of options. They typically select the channels of interest based on literature review, experience and observation. Hence, some studies only focus on the occipital-parietal area (Haxby *et al.* 2002) in the brain, such as channels Pz, P4, and P8 (Harrison and Strother 2021). Locations of these channels reflect a right hemisphere bias when viewing a face. In contrast, some other studies focus on the frontal-temporal area (Jacques *et al.* 2014) in the brain, such as FT7 and FT8 (Jacques *et al.* 2014), also related to N170. Our connectivity analysis has identified channels from both the occipital-parietal and the frontal-temporal areas, and these channels constitute a Region of Interest (ROI). It sheds light on understanding the neural mechanisms underlying face processing and further aids psychologists in automatically selecting focused channels.

## 6 Conclusion and discussion

This study proposes a framework for EEG data analysis that integrates advanced high-dimensional statistical tools to address the challenges posed by high dimensionality and temporal dependencies. The framework includes both testing changes in the mean vector and analyzing variations in the precision matrix, allowing for a comprehensive verification of the effects of stimulation. Specifically, we introduce the RIHT to test changes in the mean vector. The RIHT can be extended to handle situations where population distribution assumptions are relaxed, relying solely on the first four moment conditions. Additionally, we present a novel computational algorithm for achieving de-biased estimations in the Fused Graphical Lasso, while simultaneously conducting inference. Furthermore, we introduce a data-driven fine-tuning algorithm that automatically searches for optimal hyperparameters. Both of these methods were applied to analyze simulated data and EEG data collected from four cognitive experiments. The results demonstrate that the RIHT can accurately and sensitively detect change points, while the Fused Graphical Lasso effectively estimates and infers the functional connections among brain areas. Notably, the channel selection and dominant channel analyses conducted in this study can identify significant channels that are crucial to human cognitive ability, including PO3, PO4, Pz, P4, P8, FT7, and FT8. This further highlights the potential capabilities of our algorithms. Finally, the simulation study provides additional evidence supporting the accuracy of both the Fused Graphical Lasso and the tuning method, confirming their reliability in analyzing complex EEG data.

In summary, our goal is to confront challenges in EEG data analysis through the use of high-dimensional testing tools. It not only broadens the potential applications of high-dimensional statistics but also provides effective ways to tackle the obstacles presented by high dimensionality and temporal dependency in EEG data analysis. Although some novel algorithms have been developed to address the issues in this study, several aspects should be considered in future work. Firstly, our future work will focus on incorporating more high-dimensional tools for EEG data analysis. Even state-of-the-art methods such as deep learning will be considered in our future endeavors. Secondly, in addition to high dimensionality and temporal dependency, high-dimensional statistics should also account for other factors, such as nonlinearity, when analyzing EEG data. Thirdly, reducing the time consumption during the estimation and inference processes, as well as ensuring the tools are user-friendly and convenient to use, should also be priorities for future development. This will determine the practical usability of the statistical tools. Finally, while some intriguing channels related to human cognitive ability have been identified in both the occipital-parietal and frontal-temporal areas, further exploration and explanation are required to fully understand the implications and potential for uncovering more interesting findings. Hence, in the future, we need to collaborate with medical research institutions or hospitals to apply high-dimensional tools in diagnosing various neurological conditions, including epilepsy, insomnia, seizure detection, sleep disorders, brain injuries, emotion recognition, and BCIs.

Finally, although some interesting channels related to human cognitive ability are identified by channel selection and connectivity analysis, results between the channel selection and dominant channel analyses are different.

## Data Availability

The data used in the article are available at https://github.com/yahu911/Framework_EEG.
